# Odor Discrimination in *Drosophila*: From Neural Population Codes to Behavior

**DOI:** 10.1016/j.neuron.2013.08.006

**Published:** 2013-09-04

**Authors:** Moshe Parnas, Andrew C. Lin, Wolf Huetteroth, Gero Miesenböck

**Affiliations:** 1 Centre for Neural Circuits and Behaviour, University of Oxford, Tinsley Building, Mansfield Road, Oxford OX1 3SR, UK

## Abstract

Taking advantage of the well-characterized olfactory system of *Drosophila*, we derive a simple quantitative relationship between patterns of odorant receptor activation, the resulting internal representations of odors, and odor discrimination. Second-order excitatory and inhibitory projection neurons (ePNs and iPNs) convey olfactory information to the lateral horn, a brain region implicated in innate odor-driven behaviors. We show that the distance between ePN activity patterns is the main determinant of a fly’s spontaneous discrimination behavior. Manipulations that silence subsets of ePNs have graded behavioral consequences, and effect sizes are predicted by changes in ePN distances. ePN distances predict only innate, not learned, behavior because the latter engages the mushroom body, which enables differentiated responses to even very similar odors. Inhibition from iPNs, which scales with olfactory stimulus strength, enhances innate discrimination of closely related odors, by imposing a high-pass filter on transmitter release from ePN terminals that increases the distance between odor representations.

## Highlights

• Distances between excitatory PN (ePN) signals predict innate odor discrimination
• Silencing ePN subsets has distance-specific behavioral consequences
• Inhibitory PNs (iPNs) increase the contrast between similar odor representations
• iPNs act by high-pass filtering transmitter release from ePNs

## Teaser

Studying olfaction in *Drosophila*, Parnas et al. relate neuronal population activity to odor discrimination. The distance between projection neuron signals determines spontaneous discrimination, whereas inhibitory projection neurons improve performance by stretching this distance.

## Introduction

Most neurons involved in perceptual judgments are at least two synapses removed from sensory receptors. Therefore, psychophysical models that link perception to the physical qualities of external stimuli are black boxes: they do not account for how sensory information is encoded and how the resulting internal representations support the detection and discrimination of stimuli. Opening these black boxes has been difficult. To do so would require estimates of activity in many—ideally, all—neurons carrying perceptually relevant signals. Because sensory representations tend to be distributed over large numbers of neurons, such estimates have generally remained elusive (see Kreher et al. [2008] for a notable exception).

Here, we take advantage of the well-characterized olfactory system of fruit flies to relate knowledge of the population representations of odors to behavioral measures of odor discrimination. Flies detect odorous molecules with arrays of ∼50 types of olfactory receptor neuron (ORN) (Couto et al., 2005; Fishilevich and Vosshall, 2005) whose response spectra are determined by the expression of a single functional odorant receptor (Clyne et al., 1999; Vosshall et al., 1999; Dobritsa et al., 2003; Hallem et al., 2004). The mean spike rates evoked by 110 odorants in 24 of the ∼50 ORN types of adult flies have been measured (Hallem and Carlson, 2006; Hallem et al., 2004), providing a quantitative description of activity in approximately half of the neuronal population at the input stage of the olfactory system.

ORN axons segregate by receptor type (Gao et al., 2000; Vosshall et al., 2000) and transmit signals via separate synaptic relays, the glomeruli of the antennal lobe, to discrete classes of excitatory projection neurons (ePNs) (Jefferis et al., 2001; Stocker et al., 1990). ePN responses are saturating functions of input from cognate ORNs that scale inversely with total ORN activity (Olsen et al., 2010). Thus, a two-parameter transformation incorporating direct and total ORN activity allows estimation of mean ePN spike rates from measured ORN spike rates.

ePNs project to two brain areas: the mushroom body (MB) and the lateral horn (LH) of the protocerebrum. Innate odor-driven behaviors are thought to rely on circuits of the LH only (Heimbeck et al., 2001), whereas learned behaviors require the MBs (Heisenberg et al., 1985), whose plastic output synapses are the postulated storage sites of learned associations (Heisenberg, 2003). The MBs only receive feedforward excitation from cholinergic ePNs, whereas the LH receives parallel excitatory and inhibitory inputs via ePNs and a functionally uncharacterized group of mostly multiglomerular GABAergic inhibitory PNs (iPNs) (Jefferis et al., 2001; Lai et al., 2008; Okada et al., 2009; Tanaka et al., 2012). Inhibition has been invoked in many sensory systems as a mechanism for enhancing contrast (Barlow, 1953; Hartline et al., 1956; Kuffler, 1953), exerting gain control (Barlow, 1961; Olsen et al., 2010; Olsen and Wilson, 2008; Root et al., 2008), or binding neurons representing different stimulus features in synchrony (Gray et al., 1989; Laurent and Davidowitz, 1994; Stopfer et al., 1997). It is currently unknown whether iPNs play any of these roles.

In this study, we formulate and test a simple model of innate odor discrimination that takes as its input the estimated ePN signals projected onto the LH and generates a prediction of whether two odors are discriminated as its output. We show that the main determinant of discrimination is the distance between ePN activity patterns. Experimental manipulations of this distance have graded and predictable behavioral consequences. iPN inhibition enhances the contrast between closely related odors by imposing a high-pass filter on ePN synapses in the LH that stretches the distances between overlapping odor representations.

## Results

### Odor Discrimination from ePN Activity Patterns

**Figure 1:**
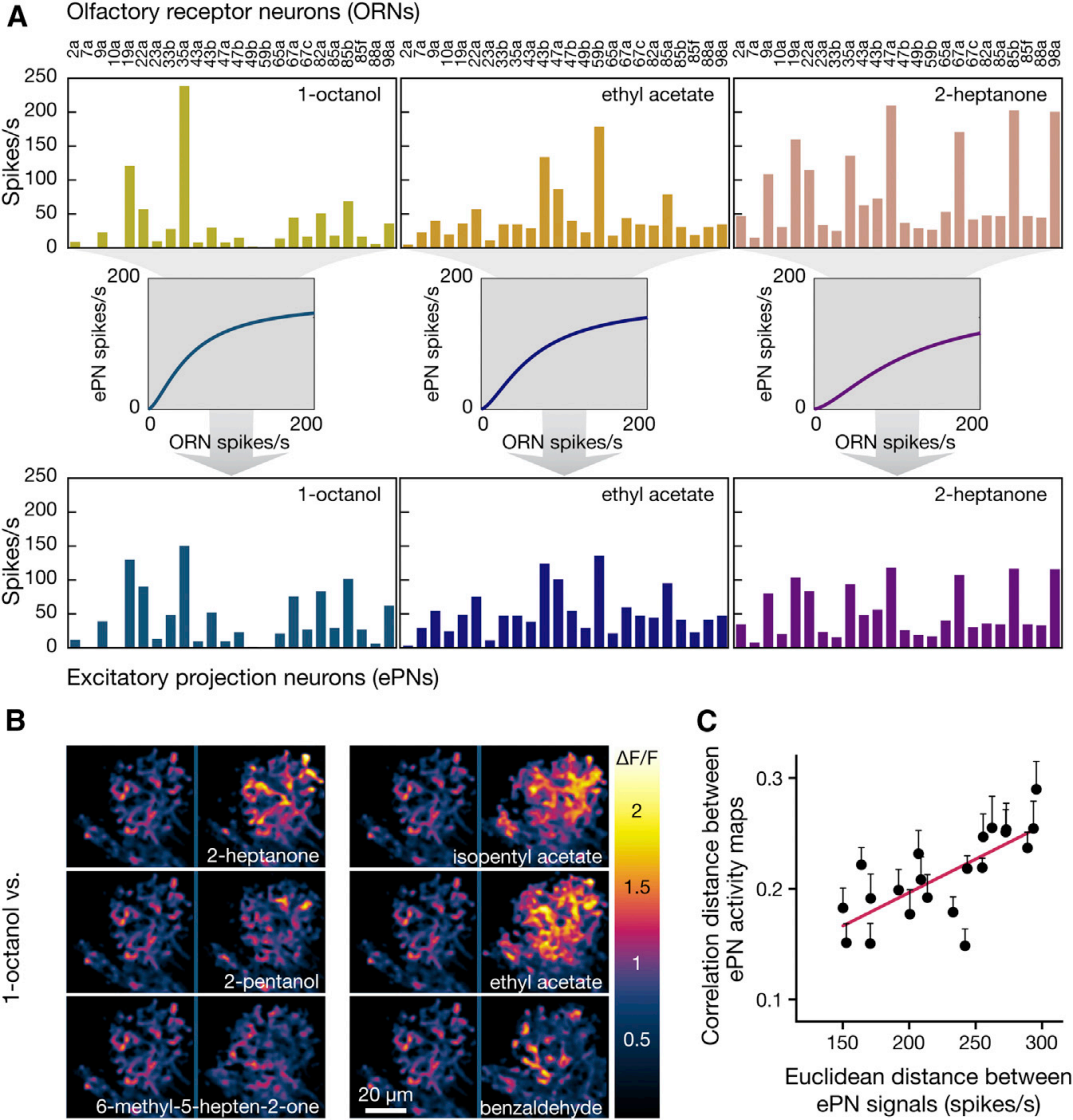
Population Representations of Odors (A) ORN (top) and ePN (bottom) representations of 1-octanol (left), ethyl acetate (center), and 2-heptanone (right). The odor responses of 24 ORN classes (expressing the odorant receptors indicated on top) were measured (Hallem and Carlson, 2006; Hallem et al., 2004). ePN responses were predicted from these measurements with the experimentally supported equation (Olsen et al., 2010):$$R_{ePN}=R_{\max}\frac{{R_{\mathrm{ORN}}}^{1.5}}{{R_{\mathrm{ORN}}}^{1.5}+\sigma^{1.5}+{\left(m/190\sum R_{ORN}\right)}^{1.5}}$$Here, $R_{ePN}$ is the firing rate of a particular class of ePN, $R_{ORN}$ is the firing rate of the cognate class of ORN, $R_{\max}$ is the maximal possible ePN firing rate, σ is a constant, and *m* is an inhibitory scaling factor. The following parameter values were used: $R_{\max}$ = 165 spikes per s, *m* = 10.63, and σ = 12 spikes per s (Luo et al., 2010; Olsen et al., 2010). The input-output relationship described by the equation is depicted graphically for the three odors. Note that the transformation differs between odors because inhibitory scaling depends on $\sum R_{ORN}$. (B) Responses in ePN projections to the LH were evoked with 5 s pulses of odors and imaged by two-photon microscopy. Flies carried *GH146-GAL4:UAS-GCaMP3* transgenes. Examples of individual responses to the six indicated odors (right) are contrasted with a common reference—the response to 1-octanol (left). The activity maps are pseudocolored according to the key on the right. (C) Correlation distances between the experimentally determined response maps are linearly related to calculated Euclidean distances between ePN activity vectors (mean ± SEM, R^2^ = 0.5334, p < 0.0001, n = 13 flies). See also Figure S1.

We considered rate code representations of odors in the ∼50 glomerular channels that constitute the front end of the fly olfactory system. Odors were denoted by vectors of ∼50 components, which indicated the mean spike frequencies in each glomerular channel. Choosing experimental odors with characterized ORN response spectra (Hallem and Carlson, 2006; Hallem et al., 2004) allowed us to assign numerical values to 24 of these ∼50 components. We termed these 24 components the ORN activity vector (Figure 1A). The corresponding ePN activity vectors were calculated by applying a saturating transformation to each ORN activity vector component plus an inhibitory scaling factor (*m*) that reflects the activation of GABAergic antennal lobe interneurons and alters the slope of the transformation as a function of total ORN activity (Olsen et al., 2010) (Figure 1A). Different glomeruli vary somewhat in their sensitivity to inhibition, but our calculations of ePN firing rates assumed a uniform scaling factor of *m* = 10.63 (Luo et al., 2010; Olsen et al., 2010). Varying *m* in the physiologically plausible range of 5 to 15 (Luo et al., 2010; Olsen et al., 2010) had little impact on our conclusions (Figure S1 available online). Because glomerular connectivity between ORNs and ePNs is 1:1 (Jefferis et al., 2001; Stocker et al., 1990), ePN activity vectors also have ∼50 components, one for the average spike frequency of each class of ePN. We could assign numerical values to 24 of these components by selecting odors with known ORN response spectra (Figure 1A).

ePN activity vectors were used to define two types of pairwise distance between odor representations (Kreher et al., 2008). The Euclidean distance is the length of the line segment connecting the tips of two activity vectors in 24-dimensional space, reflecting the distribution of firing rates across the ePN population. Cosine distance measures the angle between two activity vectors. Large cosine distances indicate that the vectors are nearly orthogonal (suggesting little overlap of the corresponding neural activity patterns), whereas small distances indicate that the vectors are nearly parallel, and the activity patterns are similar in structure but not necessarily in magnitude. The main difference between the two metrics is that Euclidean distance is sensitive to scale (i.e., the overall magnitude of firing rates), whereas cosine distance is not.

To verify that ePN activity vectors and their distances accurately reflect input to the LH, we expressed GCaMP3 (Tian et al., 2009; Wang et al., 2003) under *GH146-GAL4* control and imaged patterns of calcium influx into ePN axonal branches in the LH (Figure 1B). Distances between ePN activity vectors explained more than 50% of the observed variation in the structure of these activity maps when responses to 21 odor pairs were compared across 13 individuals (Figure 1C).

**Figure 2:**
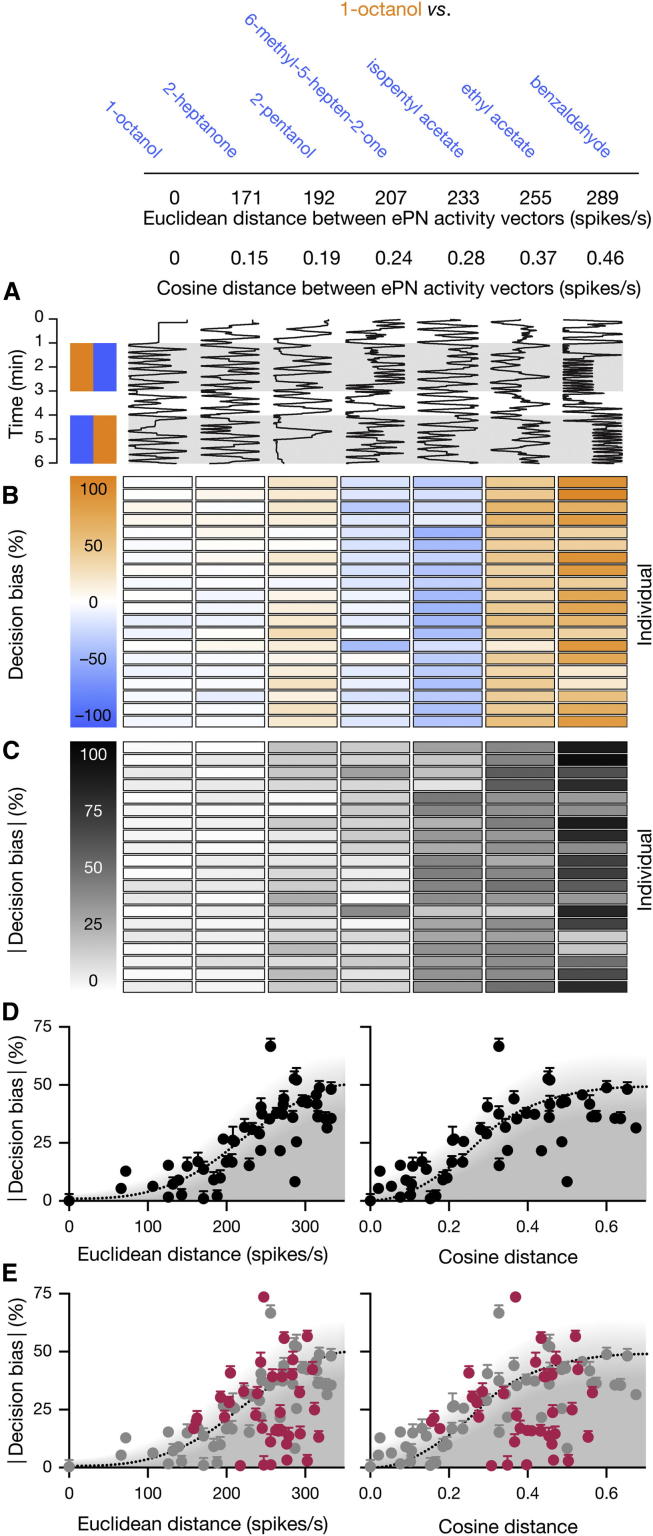
The Distance-Discrimination Function (A) Movement traces depicting the position of a single Canton-S fly in a behavioral chamber (horizontal dimension) as a function of time (vertical dimension). The same fly was tested between a common reference odor, 1-octanol (orange), and the indicated test odors (blue). The data are arranged by increasing Euclidean and cosine distances between the respective ePN activity vectors. (B) Decision bias scores of 20 Canton-S flies tested against seven odor combinations. Orange symbolizes a preference for 1-octanol, and blue symbolizes a preference for the comparison odor; the intensity of shading represents the magnitude of bias according to the key on the left. (C) Absolute magnitude of the decision bias scores depicted in (B). The intensity of shading represents the magnitude of bias according to the key on the left. (D) Absolute decision bias scores elicited by 51 odor pairs as functions of Euclidean (left) and cosine (right) distances between ePN signals (mean ± SEM, n = 40–80 flies per data point). The distance-discrimination functions (dotted lines) were obtained from least-squares logistic fits to the data; the fits were constrained to include the origin (Euclidean distance: R^2^ = 0.6577, p < 0.0001; cosine distance: R^2^ = 0.6693, p < 0.0001). Shading indicates the area bounded by the distance-discrimination function where decision bias scores are predicted to fall. See also Table S1. (E) Absolute decision bias scores elicited by 36 odors against air (red) as functions of Euclidean (left) and cosine (right) distances between ePN signals (mean ± SEM, n = 40–80 flies per data point). ePN signals in air were calculated from measured spontaneous ORN activity (Hallem and Carlson, 2006; Hallem et al., 2004). The distance-discrimination function and experimental measurements obtained in (D) are reproduced for comparison (gray). See also Table S2 and Figure S2.

Behavior was analyzed by tracking individual flies in narrow, 50 mm-long chambers (Claridge-Chang et al., 2009). The left and right halves of each chamber were perfused with independently controlled odor streams whose convergence at the midpoint defined a ∼5 mm-wide choice zone. Each time a fly entered and left this choice zone, a decision was counted (Figure 2A). Choices in favor of either odor were tallied and combined into a single decision bias score. A bias of 100% indicates that a fly always chose one odor over the other; a bias of 0% signals unbiased or random choices. The measurement period was divided into two 2 min intervals, during which the left-right positions of the odorants were reversed (Figure 2A). We selected odors from the set characterized by Hallem et al. (2004) and Hallem and Carlson (2006) that would create odor pairs spanning the whole range of possible ePN distances (Table S1).

Flies made an average of 19.9 ± 8.8 decisions per 4 min measurement period (mean ± SD, n = 10,102 experiments). When the same odor was delivered to both arms of the chamber, choices were unbiased (decision bias = 0.71% ± 3.30%; mean ± SEM, n = 161 flies) (Figure 2); when different odors were presented, each odor combination elicited a characteristic bias (Table S1), which was expressed in a qualitatively similar fashion by all members of a population (Figures 2B and 2C). Therefore, the lack of a measurable bias in a population is not a consequence of averaging opposing individual preferences.

Differences in behavioral bias can arise from two sources: differences in odor discrimination and differences in odor preference. In our analysis, we conceptually separated the processes of odor discrimination and valuation. In this two-step model of odor choice, the animal must first distinguish the odors in a pair and then decide which (if any) it prefers. If it cannot distinguish the odors, it cannot express a preference. Thus, a measurable preference indicates successful discrimination. The converse is not true: a fly may be able to tell two odors apart but may choose randomly between them if it has no incentive to act on a perceived difference. In other words, our measurements cannot distinguish indiscrimination from indifference.

Bearing in mind this limitation, we searched for predictors of behavioral bias across a data set of 51 odor pairs. Although we would not expect to predict the exact level of bias for each odor combination, given that discrimination is viewed through the lens of innate preference, general trends should nevertheless emerge. For example, if discrimination between two odors required a minimal separation between the neural representations of these odors, then significant bias should become apparent only at large ePN distances. Indeed, plots of decision bias versus Euclidean or cosine distances between ePN activity vectors showed that the magnitude of bias was bounded by logistic functions of distance for both metrics (Figure 2D). Flies expressed little or no bias when the distance between the representations of two odors was small, achieved saturating levels of bias when distances were large, and tended to display intermediate bias in the transition region between plateaus (Figure 2D). The same logistic bound held irrespective of whether flies discriminated two odors or a single odor against air (Figure 2E).

Some well-separated odor-odor pairs and many odor-air pairings elicited lower-than-expected levels of bias (Figures 2D and 2E). These cases underscore that the distance-discrimination function is an upper bound; performance necessarily falls short of this bound when flies lack pronounced innate preferences for the experimental odor(s).

When odor valences were measured individually against air and subtracted in order to generate pairwise preference distances (Figure S2 and Table S2), these preference distances generally predicted the sign of the behavioral bias, but not necessarily its magnitude (Figure S2). Indeed, our data set contains several examples of odors that generated large and opposite biases when tested individually against air but masked each other completely when paired. Hexyl acetate is a strong attractant with a bias score of 46.6%, and 2-heptanone is a weak repellant with a bias score of –15.9%; when the two odors were tested against each other, the decision bias vanished (2.6%). Similarly, isopentyl acetate is a strong attractant with a bias score of 42.4%, and ethyl butyrate is a weak repellant with a bias score of –14.6%; when these odors were tested against each other, the bias score dropped to 2.1%. The two-step model of odor choice suggests a likely explanation: if flies fail to discriminate two odors, then they are unable to attach preference selectively no matter how pronounced the preferences for the individual odors. Consistent with this interpretation, the distances between the ePN activity vectors of these odor pairs map to the bottom plateau of the distance-discrimination function (Tables S1 and S2).

### Experimental Manipulation of Distance-Based Discrimination

**Figure 3:**
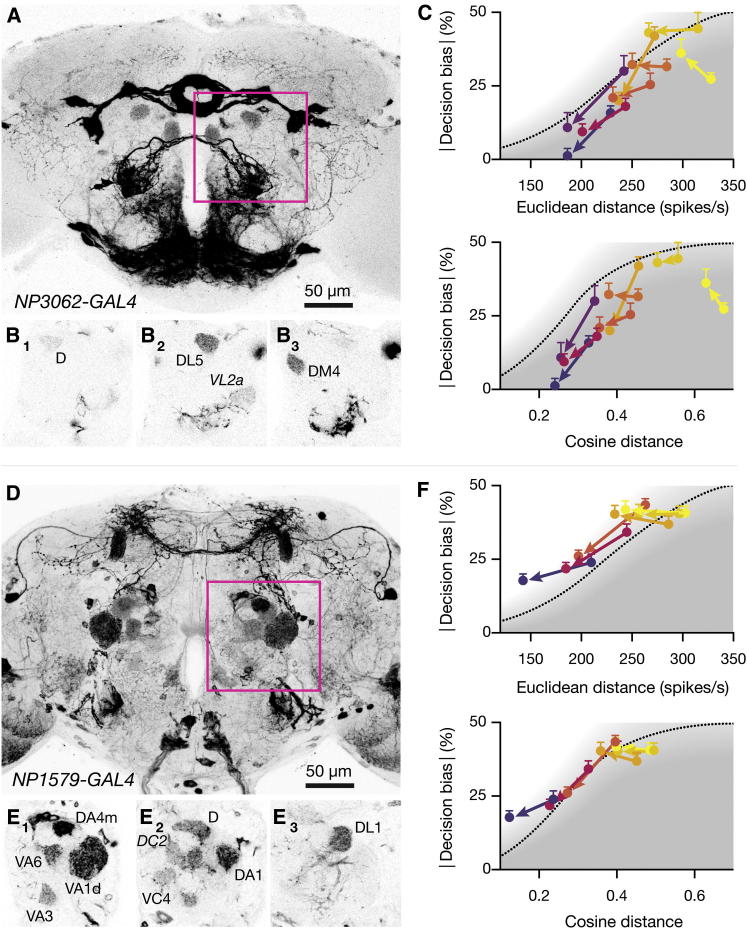
Experimental Manipulations of Distance-Based Discrimination (A) Maximum intensity projection of 117 confocal sections (1.5 μm) through the central brain of a fly carrying *NP3062-GAL4:UAS-mCD8-GFP* transgenes. (B) Single confocal sections from anterior (B_1_) to posterior (B_3_) of the antennal lobe region indicated in (A). (C) Absolute decision bias scores of flies carrying *NP3062-GAL4:UAS-shi*^*ts1*^ transgenes as functions of Euclidean or cosine distances between ePN activity vectors (mean ± SEM, n = 30–40 flies per data point). For each odor pair, colored arrows indicate the behavioral change caused by shifting flies from the permissive to the restrictive temperature. The distance-discrimination functions of WT flies, obtained in Figure 2D, are reproduced for reference. The decision bias scores of *NP3062-GAL4:UAS-shi*^*ts1*^ flies differ significantly between the permissive and restrictive temperatures, as predicted from the reduction in ePN distances (p = 0.0109 and 0.0411 for Euclidean and cosine distance, respectively; F test). (D) Maximum intensity projection of 111 confocal sections (1.5 μm) through the central brain of a fly carrying *NP1579-GAL4:UAS-mCD8-GFP* transgenes. (E) Single confocal sections from anterior (E_1_) to posterior (E_3_) of the antennal lobe region labeled in (D). (F) Absolute decision bias scores of flies carrying *NP1579-GAL4:UAS-shi*^*ts1*^ transgenes as functions of Euclidean or cosine distances between ePN activity vectors (mean ± SEM, n = 40 flies per data point). For each odor pair, colored arrows indicate the behavioral change caused by shifting flies from the permissive to the restrictive temperature. The distance-discrimination functions obtained in Figure 2D are reproduced for reference. The decision bias scores of *NP1579-GAL4:UAS-shi*^*ts1*^ flies differ significantly between the permissive and restrictive temperatures, as predicted from the reduction in ePN distances (p < 0.0001 for Euclidean and cosine distances; F test). See also Table S3 and Figure S3.

If performance is determined by the distance between ePN activity vectors, then the consequences of experimental manipulations that alter this distance should be predicted by the distance-discrimination function. To test this notion, we reversibly blocked synaptic transmission in subsets of ePNs by expressing a dominant-negative, temperature-sensitive dynamin mutant (shi^ts1^) (Kitamoto, 2001). Two enhancer trap lines provided genetic access to defined groups of ePNs: *NP3062-GAL4* (Olsen et al., 2010; Tanaka et al., 2012) labels ePNs innervating glomeruli DL5 and DM4 (for which ORN activity data are available). The line also shows weak expression in ePNs innervating D and VL2a (Figures 3A and 3B). The response spectra of ORNs projecting to DL5 and DM4 (Hallem and Carlson, 2006; Hallem et al., 2004) suggest that silencing the cognate ePNs will significantly reduce the distances between dimethylsulfide and several other odors (Table S3). The line *NP1579-GAL4* (Tanaka et al., 2012) drives expression in ePNs innervating glomeruli DA4m, DL1, VC4, VA6, and VA1d (for which ORN activity data are available) as well as D, DA1, VA3, and DC2 (Figures 3D and 3E). Judging from published ORN response spectra (Hallem and Carlson, 2006; Hallem et al., 2004), distances between acetophenone and several other odors depend heavily on activity in glomeruli DA4m, DL1, VC4, VA6, and VA1d (Table S3).

Using dimethylsulfide and acetophenone as common reference odors, we selected comparison odors in order to cover a range of distances along the distance-discrimination function (Figures 3C and 3F; Table S3). Silencing the genetically targeted ePNs shifts all data points to the left, reflecting a general reduction of distances (Figures 3C and 3F; Table S3). The expected behavioral consequences of this shift depend on where a particular odor pair lies on the distance-discrimination function. Odor pairs that sit comfortably on the top plateau will simply translate leftward but remain on the plateau; in these cases, the loss of signal from part of the ePN ensemble is predicted to be behaviorally neutral. In contrast, odor pairs that lie near the edge of the plateau or along the slope of the distance-discrimination function will move not only to the left but also slide downward; in these cases, the partial loss of ePN output is predicted to reduce bias. Consistent with these predictions, the magnitude of the behavioral change generated by silencing subsets of ePNs depended not only on the overall reduction in distance between ePN activity vectors but also on where the original distance fell on the distance-discrimination function (Figures 3C and 3F).

Each of the enhancer trap lines used in these experiments also drives expression in neurons that have not been linked to innate odor responses, such as cells of the ellipsoid body and the subesophageal ganglion (Figure 3A) or the MB output neuron MB-V2a and the dorsal-anterior-lateral neuron (Figure 3D). Two observations run counter to a role of these neurons. First, silencing synaptic output throughout the *NP3062-GAL4* or *NP1579-GAL4* expression domains causes similar behavioral phenotypes. The only neuronal elements common to both domains are ePNs (Figures 3A and 3D). Second, the distance-discrimination function, which only takes ePN activity into account, quantitatively predicts the severity of the behavioral phenotypes for all combinations of enhancer trap line and odor pairing, including the cross controls of *NP3062-GAL4* with acetophenone pairs and *NP1579-GAL4* with dimethyl sulfide pairs (Figures 3C, 3F, and S3; Table S3).

### Innate versus Learned Discrimination

To determine whether a similar distance-discrimination function also applies to learned behavior, we tested animals on the two-alternative forced-choice task after training. Here, the 4 min measurement period was preceded by a training session during which a 1 min presentation of the innately less aversive odor was followed by a 1 min presentation of the innately more aversive odor with electric shock (Claridge-Chang et al., 2009).

**Figure 4:**
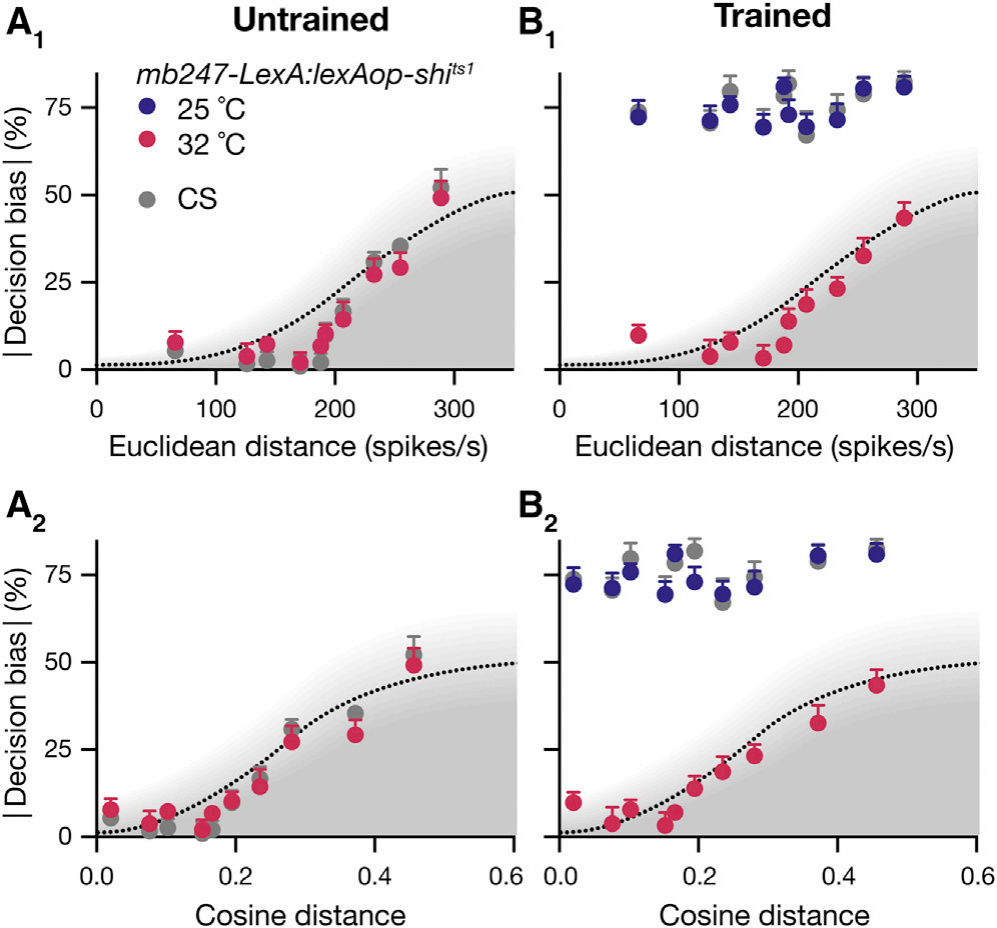
Innate versus Trained Discrimination Absolute decision bias scores of flies carrying *mb247-LexA:lexAop-shi*^*ts1*^ transgenes (mean ± SEM, n = 40–60 flies per data point) as functions of Euclidean or cosine distances between ePN activity vectors. The distance-discrimination functions obtained in Figure 2D are reproduced for reference. (A) Innate discrimination at the restrictive temperature (32°C) when synaptic output from KCs is blocked. Absolute decision bias scores as functions of Euclidean (A_1_) or cosine (A_2_) distances between ePN activity vectors (mean ± SEM, n = 30–60 flies per data point). (B) Avoidance of the innately more aversive odor in a pair was reinforced during a 1 min cycle of electric shock training at the permissive temperature (25°C). After a 15 min rest interval, odor discrimination was analyzed at either the permissive or the restrictive temperature when synaptic output from KCs is intact (25°C, blue) or blocked (32°C, red). Absolute decision bias scores as functions of Euclidean (B_1_) or cosine (B_2_) distances between ePN activity vectors (mean ± SEM, n = 30–60 flies per data point). See also Table S4 and Figure S4.

Trained decision bias was no longer bounded by a logistic function of distance between ePN signals; instead, it remained virtually constant at 73.5% ± 1.6% (mean ± SEM), even for the two odors separated by the shortest distance among all 5,995 possible pairs in the panel (Figure 4; Table S4). Given that innate and learned behavior are thought to be controlled by separate brain regions (the LH and MB, respectively) (Heimbeck et al., 2001), differences in innate and learned discrimination may arise because the LH and MB use different odor-coding formats, the MB supporting finer discrimination than the LH. If untrained flies disregarded information encoded in the MB and made use of LH signals exclusively, then they would display only coarse discrimination.

To test this conjecture, we expressed *lexAop-shi*^*ts1*^ under *mb247-LexA* control in Kenyon cells (KCs), the principal intrinsic neurons of the MBs. Switching off the efferent synapses of KCs during testing occluded the effects of learning: the decision bias of trained flies now followed the same distance-discrimination function as that of untrained flies (Figure 4B). Both parental control strains showed wild-type (WT) performance at the elevated temperature (Figure S4). Thus, preventing the retrieval of memory in trained animals re-exposed their innate behavioral state. In contrast, blocking KC output in untrained flies had no discernible behavioral consequence; the distance-discrimination functions of untrained animals with intact and blocked MB output overlapped precisely (Figure 4A). We conclude that flies use two parallel odor representations in a state-dependent manner: they rely on the LH alone in the untrained state and engage the MB only after training. Failures of untrained flies to discriminate behaviorally between odors that are separated by small ePN distances, despite strong and opposing preferences to each odor alone, must reflect the coarse grain of odor representation in the LH and a lack of incentive to draw on the fine discrimination system of the MB.

### Inhibition by GABAergic PNs Enhances Innate Odor Discrimination

**Figure 5:**
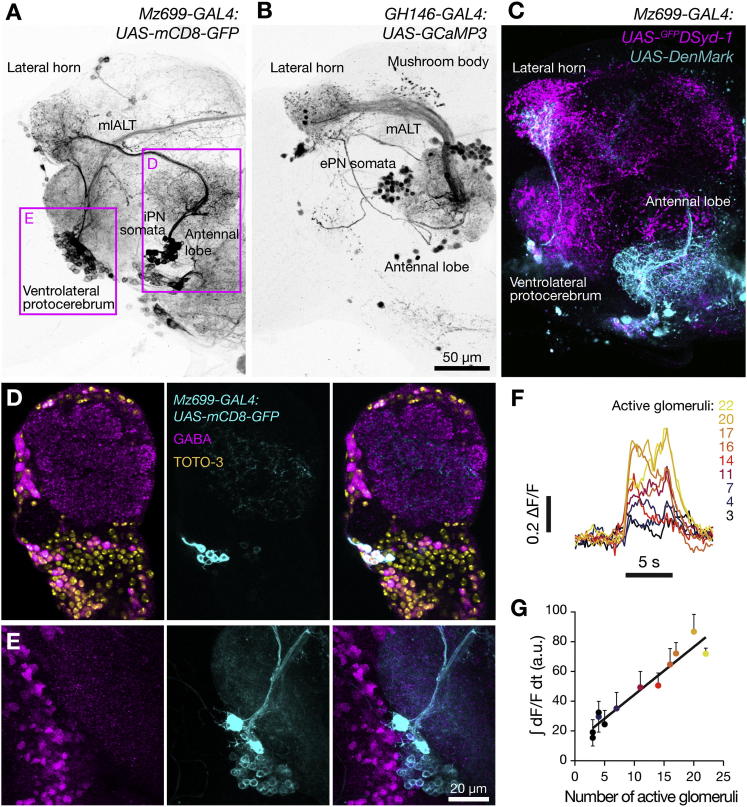
Excitatory and Inhibitory Projections from the Antennal Lobe to the MB and LH (A) Maximum intensity projection of 119 confocal sections (1.5 μm) through the central brain of a fly carrying *Mz699-GAL4:UAS-mCD8-GFP* transgenes. The *Mz699* enhancer element labels ∼39 ventral iPNs (D) and ∼86 cells in the vlpr (E). (B) Maximum intensity projection of 113 confocal sections (1.5 μm) through the central brain of a fly carrying *GH146-GAL4:UAS-GCaMP3* transgenes. The *GH146* enhancer element labels ∼90 mostly excitatory dorsal and lateral PNs. (C) Maximum intensity projection of 67 confocal sections (1.5 μm) through the central brain of a fly carrying *Mz699-GAL4:UAS-*^*GFP*^*DSyd-1;UAS-DenMark* transgenes. ^GFP^DSyd-1 (magenta) labels presynaptic terminals. Fluorescence in the LH originates mainly from iPN axons, whereas signal in the vlpr arises from ipsi- and/or contralateral projections of vlpr neurons. The vlpr cells may also elaborate presynaptic sites in the LH, but these are obscured by the strong iPN signal. DenMark (cyan) labels putative dendritic regions. Although iPN dendrites are found exclusively in the antennal lobes, vlpr neurons receive their main input in the LH. Faint DenMark labeling suggests additional weak dendritic sites of vlpr cells in the vlpr. See Movie S1 for the complete image stack. (D and E) Confocal sections through the central brain of a fly carrying *Mz699-GAL4:UAS-mCD8-GFP* transgenes, after immunostaining against GABA (magenta, left column) and GFP (cyan, center column); colocalization of both markers results in white structures in the overlay images on the right. In some images, nuclei are counterstained with TOTO-3 (yellow). The approximate positions of the imaged areas are indicated in (A). The images were acquired and are displayed at different photomultiplier gain and contrast settings. (D) An individual confocal section (1 μm) shows GABAergic iPNs. See Movie S2 for the complete confocal image stack. (E) A maximum intensity projection of 70 confocal sections (1 μm) demonstrates the absence of GABA staining in vlpr neurons. See Movie S3 for the complete confocal image stack. (F and G) Two-photon imaging of odor-evoked calcium transients in flies carrying *MZ699-GAL4:UAS-GCaMP3* transgenes. (F) Single-trial responses of iPN axons to 5 s pulses of nine different odors (black bar). The traces, which were recorded in the same fly, are aligned to the time of odor onset and color-coded according to the number of glomeruli an odor activates. (G) Integrated fluorescence transients (area under the fluorescence trace during a 5 s odor pulse) in iPN axons as a function of the number of glomeruli an odor activates (mean ± SEM, R^2^ = 0.9278, p < 0.0001, n = 11 flies per data point). See also Figure S5 and Movies S1–S3.

The enhancer trap line *Mz699-GAL4* (Lai et al., 2008; Okada et al., 2009) labels 39.3 ± 0.5 GABA-positive PNs (mean ± SD, n = 4 hemispheres) located in a cluster at the ventral face of the antennal lobes (Figures 5A and 5D; Movies S1 and S2). Most of these GABAergic iPNs extend dendrites into multiple glomeruli (Lai et al., 2008; Tanaka et al., 2012) and project their axon via the mediolateral antennal lobe tract (mlALT, formerly the medial antennocerebral tract or mACT) to the LH (Figures 5A and 5C; Movie S3) (Lai et al., 2008; Tanaka et al., 2012). In contrast, the vast majority of the ∼90 ePNs marked by *GH146-GAL4* possess uniglomerular dendrites and project via the medial antennal lobe tract (mALT, formerly the inner antennocerebral tract or iACT) to both the MB and LH (Figure 5B) (Tanaka et al., 2012).

Because iPN dendrites sample many glomerular channels, odor-evoked iPN activity, like that of multiglomerular local neurons (Olsen et al., 2010), might scale with overall excitation in the olfactory system. To test this idea, we expressed GCaMP3 under *Mz699-GAL4* control and imaged the bundle of iPN axons innervating the LH as a proxy for iPN output. As expected, the time integral of odor-evoked fluorescence changes correlated with two estimates of olfactory stimulus strength (Figures 5F, 5G, and S5A): the sum of spike rates across the 24 characterized ORN classes (Figure S5A); and the number of active glomerular channels, which was determined by thresholding ORN spike rates at 30 Hz (Figure 5G; see Figure S5B for a justification of threshold). The odor responses of iPNs were predicted more accurately by the number of active glomerular channels than by the summed spike rates in these channels (Figures 5G and S5B). This result can be understood as a consequence of short-term depression at ORN synapses (Kazama and Wilson, 2008), which clips excitation to iPNs when only a few ORN classes are highly active but generates an effective drive when many ORN types fire at moderate rates.

**Figure 6:**
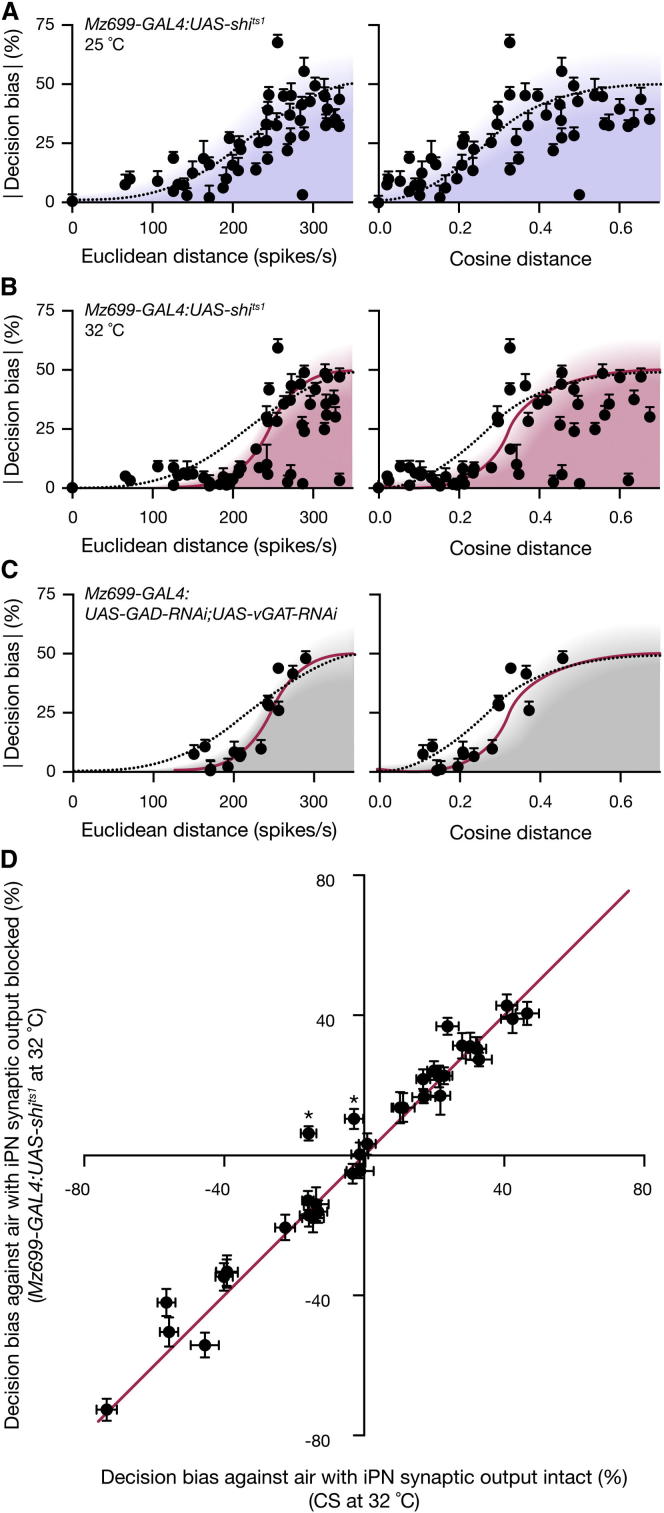
iPN Inhibition Facilitates Odor Discrimination (A) Absolute decision bias scores of flies carrying *Mz699-GAL4:UAS-shi*^*ts1*^ transgenes as functions of Euclidean or cosine distances between ePN activity vectors at the permissive temperature of 25°C when iPN-mediated inhibition is intact (mean ± SEM, n = 40–60 flies per data point). The distance-discrimination functions obtained in Figure 2D are reproduced for reference. The distance-discrimination functions of flies carrying *Mz699-GAL4:UAS-shi*^*ts1*^ transgenes are identical to those of WT flies at the permissive temperature (p = 0.9895 and 0.9813 for Euclidean and cosine distance, respectively; F test). (B) Absolute decision bias scores of flies carrying *Mz699-GAL4:UAS-shi*^*ts1*^ transgenes as functions of Euclidean or cosine distances between ePN activity vectors at the restrictive temperature of 32°C when iPN-mediated inhibition is blocked (mean ± SEM, n = 40–60 flies per data point). The distance-discrimination functions in the absence of inhibition (red lines) were obtained from least-squares logistic fits to the data; the fits were constrained to include the origin (Euclidean distance: R^2^ = 0.6779, p < 0.0001; cosine distance: R^2^ = 0.5538, p < 0.0001). The distance-discrimination functions obtained in Figure 2D (dotted lines) are reproduced for reference. The distance-discrimination functions of flies carrying *Mz699-GAL4:UAS-shi*^*ts1*^ transgenes differ significantly between the permissive and restrictive temperatures (p = 0.0058 and 0.0097 for Euclidean and cosine distance, respectively; F test). See also Table S5 and Figure S6A. (C) Absolute decision bias scores of flies carrying *Mz699-GAL4:UAS-GAD-RNAi;UAS-vGAT-RNAi* transgenes as functions of Euclidean or cosine distances between ePN activity vectors (mean ± SEM, n = 40–60 flies per data point). RNA-mediated interference with the expression of GAD and vGAT in iPNs changes the distance-discrimination functions in the same manner as blocking iPN synaptic output (see B; p = 0.3326 and 0.8711 for Euclidean and cosine distance, respectively; F test). The distance-discrimination functions of flies carrying *Mz699-GAL4: UAS-vGAT RNAi, UAS-GAD RNAi* transgenes differ significantly from those of WT flies (p = 0.0043 and 0.0263 for Euclidean and cosine distance, respectively; F test). See also Table S6 and Figure S6A. (D) Odor preferences against air of Canton-S flies and of flies carrying *Mz699-GAL4:UAS-shi*^*ts1*^ transgenes at the restrictive temperature are identical (mean ± SEM, n = 40–60 flies per data point) except for pentyl acetate and 2-heptanone (^∗^p < 0.05; Bonferroni-corrected t test). See also Table S2 and Figures S2A and S6B.

Interference with synaptic transmission from iPNs via the expression of shi^ts1^ under *Mz699-GAL4* control altered the behavioral responses to odors in a subtle but characteristic way. Blocking iPN output preserved the sigmoid shape of the distance-discrimination function but displaced the foot of the curve to the right, compressing the range of distances that elicited a behavioral bias (Figures 6A and 6B; Table S5). Thus, iPN output facilitates the discrimination of closely related ePN activity patterns. Inhibition had no general effect on the attractiveness or repulsiveness of odors determined individually against air (Figures 6D and S2A; Table S2).

However, the interpretation of this experiment is complicated by the activity of the *Mz699* enhancer element in a group of 86 ± 1 neurons (mean ± SD, n = 4 hemispheres) in the ventrolateral protocerebrum (vlpr) whose dendrites enter the LH (Figures 5A and 5C; Movie S1). Because shi^ts1^ imposes a transmission block on all neurons in which it is expressed in stoichiometric amounts (Kitamoto, 2001), we cannot ascribe the behavioral phenotype with confidence to a loss of iPN inhibition; impairment of vlpr neurons remains a viable alternative. To eliminate this alternative, we manipulated the capacity to synthesize and package the transmitter GABA, which is unique to GABAergic neurons, instead of ubiquitous synaptic vesicle recycling machinery. Because iPNs are the only prominent GABAergic cells within the *Mz699* domain (Figures 5D and 5E; Movies S2 and S3), they are also the principal targets of RNAi against the GABA-biosynthetic enzyme glutamic acid decarboxylase (GAD) and the vesicular GABA transporter (vGAT). Inducible *Mz699-GAL4*-directed knockdown of GAD and vGAT precisely replicated the behavioral phenotype observed after blocking synaptic output (Figure 6C; Table S6). Thus, the consequences of silencing iPNs and vlpr neurons are accounted for in full by a loss of iPN inhibition (Figures 6B and 6C).

An important corollary of this result is that the non-GABAergic vlpr neurons are not required for odor discrimination in our assay. Consistent with this conclusion, vlpr neurons respond selectively to pheromones and not general odors (Liang et al., 2013). Both ePN and iPN projections innervate a larger LH domain than vlpr neuron dendrites (Figures 5A–5C), suggesting that still unidentified LH neurons mediate general odor responses.

### An Inhibitory High-Pass Filter of ePN Output

The distance-discrimination model suggests that iPN inhibition stretches the distances between ePN activity vectors in order to enhance discrimination. This is not a trivial transformation to accomplish. Proportional inhibition of ePN spike rates, for example, would inevitably shrink Euclidean distances while leaving cosine distances unaltered. However, calculations and several precedents (Legenstein and Maass, 2008; Luo et al., 2010; Olsen et al., 2010) suggest that the desired separation of ePN activity vectors could be achieved through inhibition that selectively blocks low-frequency spike trains. We call this form of inhibition a “high-pass filter” because it allows high-frequency spike trains to pass (Abbott and Regehr, 2004). Similar phenomena have also been termed input gain control (Olsen et al., 2010) or input division (Mysore and Knudsen, 2012).

**Figure 7:**
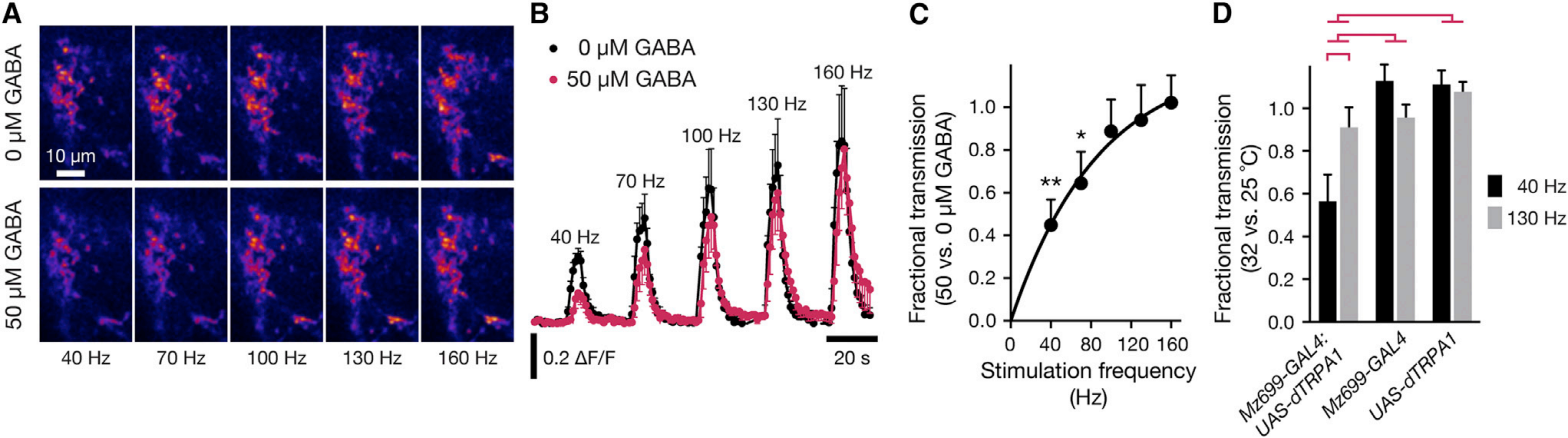
iPN Inhibition Imposes a High-Pass Filter on ePN Synaptic Output (A) Raw two-photon images of spH fluorescence in ePN projections to the LH at the indicated stimulation frequencies, in the absence (top) or presence (bottom) of 50 μM GABA. ePN axons were stimulated for 5 s by passing 1 ms pulses of current via an extracellular electrode. (B) Average spH fluorescence changes in ePN projections to the LH, evoked by electrical stimulation at the indicated frequencies, in the absence (black) or presence (red) of 50 μM GABA (mean ± SEM, n = 5 flies). (C) Average ratio of integrated spH fluorescence transients (areas under the fluorescence traces during 5 s electrical stimulation) in the presence and absence of 50 μM GABA (mean ± SEM, n = 5 flies). The ratios of ΔF/F at 0 versus 50 μM GABA differ across frequencies (p < 0.0001; one-way repeated measures ANOVA). Asterisks indicate significant differences between the presence and absence of 50 μM GABA at specific frequencies (^∗^p < 0.05, ^∗∗^p < 0.005; paired t test). (D) Thermally evoked iPN activity has a similar effect on ePN synaptic release as bath application of 50 μM GABA. Flies carried *GH146-QF*, *QUAS-spH*, *Mz699-GAL4*, and *UAS-dTRPA1* transgenes. spH fluorescence changes were measured at two electrical stimulation frequencies (40 and 130 Hz) while flies were held at 25°C and 32°C. Columns depict the ratios of the integrated spH fluorescence transients (areas under the fluorescence traces during 5 s electrical stimulation trains) between 32°C and 25°C (mean ± SEM, n = 7–8 flies). A ratio of 1 indicates no effect of thermally evoked iPN activity on ePN synaptic release, whereas a ratio <1 indicates that iPN activity inhibits ePN output. Red brackets denote significant differences (p < 0.05, with Bonferroni-corrected paired t tests to compare the 32°C:25°C ratios at 40 versus 130 Hz within genotypes and one-way ANOVA with a Tukey-Kramer post hoc test to compare the ratios of the 32°C:25°C ratios at 40 versus 130 Hz across genotypes).

To test whether input gain control might be realized in the LH, we measured synaptic vesicle release from ePN terminals expressing synapto-pHluorin (spH) (Miesenböck et al., 1998; Ng et al., 2002) under *GH146-GAL4* control in the absence or presence of 50 μM bath-applied GABA (Figures 7A and 7B). ORN input was abolished by removing both antennae, and ePN axons were stimulated by passing 1 ms pulses of current via an extracellular electrode attached to the mALT. Electrical instead of odor stimulation allowed us to control spike rates uniformly across the ePN population and isolate the presynaptic effects of GABA in the LH from its known actions on odor-evoked activity in the antennal lobe (Olsen et al., 2010; Olsen and Wilson, 2008; Root et al., 2008; Wilson and Laurent, 2005). Two-photon imaging revealed rapid, transient increases in spH fluorescence during electrical stimulation (Figures 7A, 7B, and S7). These changes reflect cycles of synaptic vesicle exo- and endo-cytosis, during which the protonation-dependent quenching of spH fluorescence is temporarily relieved (Miesenböck et al., 1998). The average peak increase in fluorescence rose smoothly with stimulation frequency in the presence and absence of GABA, but the frequency dependence of vesicle release differed in the two conditions. In comparison to control conditions, the presence of GABA severely attenuated spH signals at low stimulation frequencies but had little effect at high frequencies (Figures 7A–7C), including when total spike number was kept constant (Figure S7). Thus, the presynaptic terminals of ePNs in the LH contain machinery that allows GABA to modulate vesicle release in the manner of a high-pass filter (Figure 7C).

To examine whether iPNs could supply modulatory GABA to ePN terminals, we expressed a *QUAS-spH* transgene under *GH146-QF* control in ePNs and a *UAS-dTRPA1* transgene under *Mz699-GAL4* control in iPNs. dTRPA1 is a transient receptor potential channel whose Ca^2+^ conductance gates open at temperatures >25°C (Hamada et al., 2008), thus stimulating iPN activity. We shifted flies between holding temperatures of 25°C and 32°C while imaging spH fluorescence during electrical stimulation of ePN axons. Like the direct application of GABA (Figure 7C), the thermal activation of iPNs had a frequency-dependent effect on ePN synaptic output (Figure 7D): transmission at 130 Hz was unaffected by iPN activity, whereas transmission at 40 Hz was roughly cut in half (Figure 7D). Thus, iPN projections to the LH regulate the transmission characteristics of ePN terminals.

**Figure 8:**
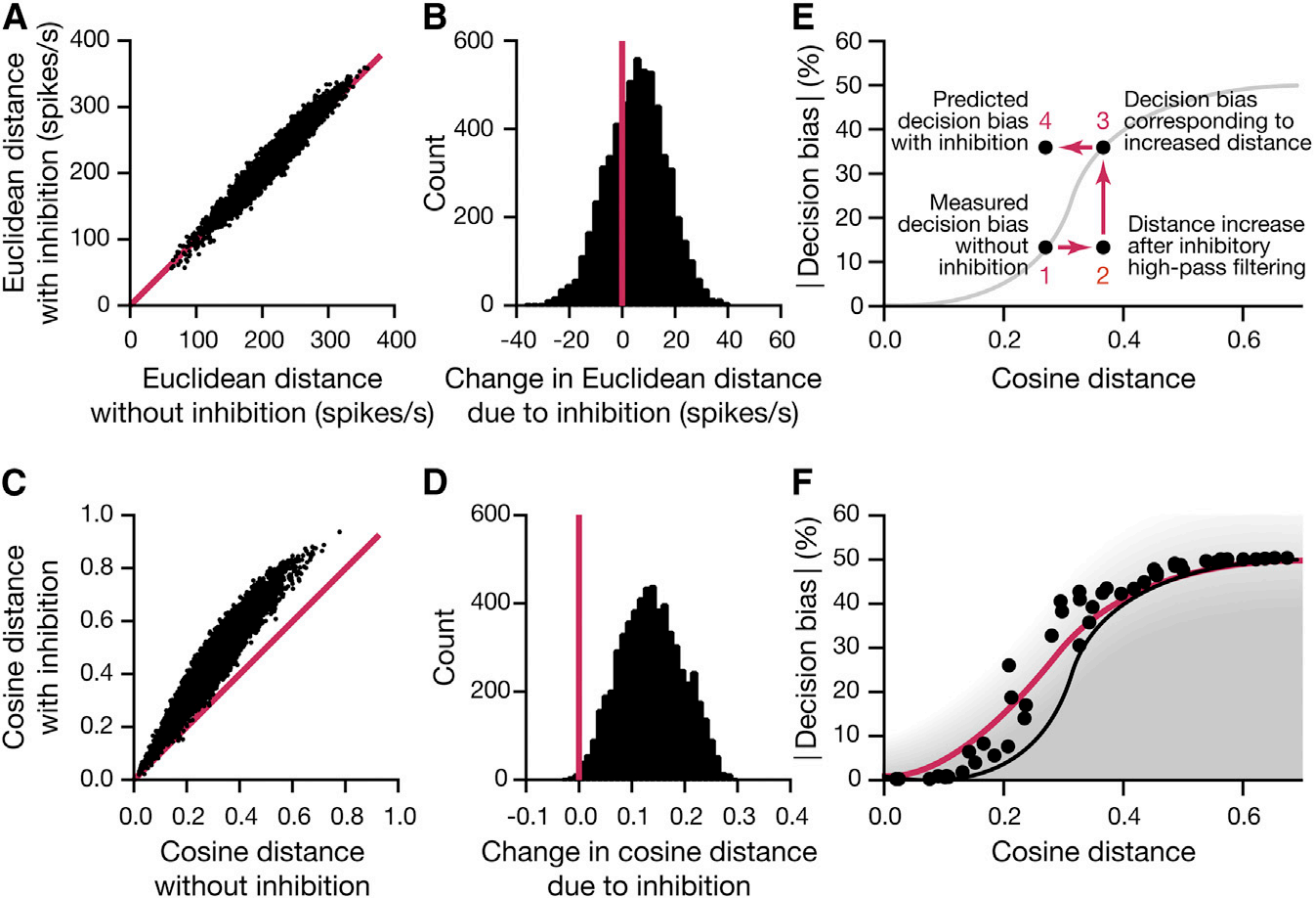
iPN Inhibition Increases ePN Distances (A and C) Euclidean (A) and cosine (C) distances between odors were calculated with the empirically derived transmission characteristics of the inhibitory high-pass filter (Figure 7C). The blocking strength of the filter was linearly adjusted according to the number of active glomeruli (Figure 5G). The scatter plot relates the 5,995 possible pairwise Euclidean distances between 110 odors after filtering to their unfiltered counterparts. (B and D) Histograms of the effect sizes of inhibitory high-pass filtering on the Euclidean (B) and cosine (D) distances between 5,995 odor pairs. The filter causes mean increases in Euclidean distance of 5.5 spikes per s (B) or in cosine distance of 0.14 (D). (E) Application of the empirically derived high-pass filter (Figure 7C) to the ePN activity vectors of two odors (point 1) stretches the cosine distance between the odors (point 2). According to the distance-discrimination model, this results in improved odor discrimination (point 3). Thus, the decision bias of WT flies with inhibition intact (point 4) is identical to the decision bias of flies in which iPN output is blocked but the distance-enhancing effect of inhibition is accounted for computationally (point 3). (F) Sequential applications of the inhibitory high-pass filter and the distance-discrimination model predict the empirical distance-discrimination function of WT flies (red line, reproduced from Figure 2D) from the empirical distance-discrimination function of flies carrying *Mz699-GAL4:UAS-shi*^*ts1*^ transgenes at the restrictive temperature of 32°C (black line, reproduced from Figure 6B).

To simulate the impact of the inhibitory high-pass filter on odor discrimination, we passed the ePN activity vectors of 110 odors (Hallem and Carlson, 2006; Hallem et al., 2004) through a filter with the empirically derived transmission characteristics (Figure 7C). Because iPN activity scales with the overall drive to the olfactory system (Figure 5G), the strength of the filter was adjusted linearly with the number of glomerular channels an odor activates. We assumed that the maximal blocking effect, corresponding to the transmission curve in 50 μM GABA (Figure 7C), is achieved when ePN spike rates in 22 of the 24 characterized glomeruli exceed 30 Hz (Figure 5G). Comparisons of all 5,995 possible pairwise distances between the filtered vectors with their 5,995 unfiltered counterparts showed that inhibition shifts the distributions of both Euclidean and cosine distances toward larger values (Figures 8A–8D). Replotting the data from Figure 2 against these increased ePN distances preserved the shape of the distance-discrimination function, only displacing it to the right (Figure S8).

Knowledge of the transmission characteristics of the inhibitory high-pass filter should enable a prediction of WT performance from the measured behavior of flies lacking the distance-enhancing effect of the filter. We attempted such a prediction as our final test of the distance-discrimination model (Figures 8E and 8F). If the principal determinant of discrimination is ePN distance, then the decision bias of WT flies with intact iPN function should be the same as the decision bias of *Mz699-GAL4:UAS-shi*^*ts1*^ flies with compromised iPN function, provided the distance-enhancing effect of inhibition is accounted for separately (Figure 8E). To do this, we applied the empirically derived high-pass filter (Figure 7C) to the odor pairs analyzed behaviorally in Figure 6B and calculated the resulting increases in distance between ePN activity vectors. Plugging the increased distances into the measured distance-discrimination function of *Mz699-GAL4:UAS-shi*^*ts1*^ flies at the restrictive temperature (Figure 8F, black line) reproduced the distance-discrimination function of WT flies at the original distances (Figure 8F, red line). Thus, presynaptic inhibition at ePN terminals in the LH explains the gain in performance within the context of the distance-discrimination model.

## Discussion

### The Distance-Discrimination Model

The experiments reported here form the basis of a distance-discrimination model of innate olfactory behavior. The central tenet of this model is that the magnitude of spontaneous responses to odors, mediated by the LH, is bounded by a logistic function of distance between the corresponding patterns of odor-evoked activity across the ePN population. The larger this difference in ePN activity is, and, therefore, the more dissimilar the neuronal signals representing the two alternatives in the choice task, the more pronounced is the behavioral bias elicited by these alternatives (Figure 2D). The distance-discrimination function is logistic, similar to many other examples in the statistical analysis of binary choices where the logistic function serves as the link between a continuous predictor variable, such as the spike rate of a neuron, and a categorical outcome, such as a decision between two alternatives.

From the viewpoint of a fly, the odor-evoked activity of its PNs provides noisy evidence from which the identity of the odors in the left and right arms of the chamber must be judged. To decide whether these odors are different or the same, the fly uses the distance between odor representations as its decision variable (Figure 2D). A decision variable quantifies the weight of evidence supporting a hypothesis (here, that the odors in the two halves of the chamber are different) over its negation (here, that the odors are the same); mathematically, the decision variable gives the log odds that the hypothesis is true (Gold and Shadlen, 2001; Good, 1985). The logistic dependence of performance on the distance between ePN activity vectors indicates that the fly decides on the weight of the sensory evidence (Good, 1985). If evidence that two odors are different is lacking (that is, if the ePN distance is small), then the fly displays indiscrimination; if the evidence is ambiguous, then the best attainable odds of correct choices are given by the distance-discrimination function; if the evidence is compelling, then performance plateaus.

The distance-discrimination model gives equal weight to signals carried by all types of ePNs and only takes average firing rates into account; there is no need to consider information encoded in timing relationships among spikes or invoke privileged receptor channels propagating signals with special behavioral significance. Although dedicated channels undoubtedly exist for mediating stereotyped responses to mating pheromones (Kurtovic et al., 2007; van der Goes van Naters and Carlson, 2007), the stress odorant CO_2_ (Suh et al., 2004), or the microbial odorant geosmin (Stensmyr et al., 2012), it remains unresolved whether innate odor responses in general reflect the activation of labeled lines that trigger hardwired behaviors (Gupta and Stopfer, 2012; Jefferis et al., 2007; Knaden et al., 2012; Semmelhack and Wang, 2009). In our hands, experimental manipulations that silence subsets of ePNs have graded, context-specific behavioral consequences; the same manipulation affects responses to different odor pairs differently, and effect sizes depend not only on the overall change but also on the initial distance between the respective ePN activity vectors (Figure 3). This finding suggests that innate responses to odors draw on many glomerular channels and not just a select few. If attraction and aversion to our test stimuli were driven by signals in single dedicated channels, as has been suggested for some generalist odors (Semmelhack and Wang, 2009), then the consequences of manipulating ePN output should be all or nothing: eliminating transmission in an essential channel should abolish all behavioral bias, whereas interference with a nonessential channel should have no effect. The data in Figure 3 are difficult to reconcile with such a scenario.

### Mechanisms for Improving Stimulus Separation

The two brain regions targeted by ePNs employ distinct mechanisms for improving the contrast of the activity patterns projected onto them: expansion recoding in the MB and input gain control in the LH.

Olfactory signals from ∼150 ePNs are projected onto ∼2,500 KCs and an unknown, though, in all likelihood, significantly smaller, number of intrinsic LH neurons. Thus, the MB recodes compact, dense ePN activity patterns into a much larger ensemble of KCs (Jortner et al., 2007). Consistent with the idea that expansion recoding facilitates stimulus separation (Albus, 1971; Marr, 1969), the significant performance benefit of training can be attributed entirely to the MBs, given that interrupting transmission through the MB loop occludes the effects of learning (Figure 4B). The finding that spontaneous behavioral bias is identical regardless of whether MB output is blocked or intact (Figure 4A) indicates that untrained flies do not access discrimination information that is presumably always available in the MB.

In the LH, a group of ∼40 GABAergic iPNs provide presynaptic inhibition to ePN terminals (Figures 5, 6, and 7). iPN output improves innate performance when the distance between two odor representations is small, but it has no effect in the plateau regions of the distance-discrimination function (Figures 6B and 6C). Consistent with previous results (Legenstein and Maass, 2008; Luo et al., 2010; Olsen et al., 2010), we find that input gain control, which selectively attenuates low-frequency ePN signals but transmits high-frequency signals in full, can amplify large differences in firing rate and thereby increase the separation between two sensory images (Figure 8). Because the high-pass filter must operate on the individual components of the ePN activity vector in order to achieve the desired effect, the likely target of inhibition in the LH is the presynaptic terminals of ePNs, which each represent a single activity vector component rather than the postsynaptic dendrites of intrinsic LH neurons, which may combine several activity vector components after synaptic integration (Gupta and Stopfer, 2012; Luo et al., 2010). Our experimental evidence supports all aspects of this mechanism. We find that GABA modulates synaptic vesicle exocytosis at ePN terminals in the LH (Figures 7A and 7B); we show that GABAergic modulation converts these terminals to high-pass filters (Figure 7C), and we identify iPN projections as the source of modulatory GABA (Figure 7D).

The arrangement of parallel ePN and iPN projections to the LH appears to result in a tunable filter whose transmission characteristics adjust to the level of activity in the olfactory system (Figures 5G and 7). What might be the reason for scaling the strength of iPN inhibition with the overall level of ORN input? One possible advantage is to balance competing demands of sensitivity and contrast. At low levels of ORN input, ePN activity would be weak; therefore, in order to detect odors with maximal sensitivity, iPN activity would be curbed to allow the unimpeded transmission of low-frequency spike trains by ePN terminals. Only at higher levels of ORN input, where sensitivity to ePN spikes is a less pressing need, would the iPN high-pass filter be engaged in order to block the transmission of low-frequency spike trains and thereby enhance discrimination.

## Experimental Procedures

### Fly Strains

Fly strains (see the Supplemental Experimental Procedures) were raised on cornmeal agar under a 12 hr light/12 hr dark cycle and studied 8–10 days posteclosion. Strains were cultivated at 25°C unless they expressed temperature-sensitive gene products (shi^ts1^, GAL80^ts^, and dTRPA1); in these cases, the experimental animals and all relevant controls were grown at 21°C. To block synaptic transmission with shi^ts1^ (Kitamoto, 2001), we incubated experimental and control animals at 32°C for 15 min before the start of a behavioral experiment and maintained them at the elevated temperature throughout. To derepress the expression of RNAi with GAL80^ts^ (McGuire et al., 2003), we incubated experimental and control animals at 31°C for 24 hr. Subsequent behavioral experiments were performed at 32°C.

### Behavioral Analysis

Behavioral experiments were performed in a custom-built, fully automated apparatus (Claridge-Chang et al., 2009) at 32°C unless stated otherwise (see the Supplemental Experimental Procedures). Data were analyzed in MATLAB 2009b (MathWorks), SigmaPlot 12.5 (Systat Software), and Prism 6 (GraphPad).

### Functional Imaging

ePN or iPN projections to the LH were imaged by two-photon laser scanning microscopy (Ng et al., 2002; Wang et al., 2003). Cuticle and trachea in a window overlying the LH were removed, and the exposed brain was superfused with carbogenated solution (95% O_2_ and 5% CO_2_) containing 103 mM NaCl, 3 mM KCl, 5 mM trehalose, 10 mM glucose, 26 mM NaHCO_3_, 1 mM NaH_2_PO_4_, 3 mM CaCl_2_, 4 mM MgCl_2_, and 5 mM N-Tris (TES) (pH 7.3). Odors at 10^−2^ dilution were delivered by switching mass-flow-controlled carrier and stimulus streams (CMOSens performance line, Sensirion) via software-controlled solenoid valves (the Lee Company). Flow rates at the exit port of the odor tube were 0.5 l per min.

Basal plasma membrane fluorescence of ePNs expressing spH was used to target a suction electrode to the mALT. Spikes were elicited with 1 ms pulses of current (10–30 μA) with a DS3 stimulus isolator (Digitimer). For thermal stimulation of iPNs expressing dTRPA1, the superfusion solution was heated with a closed-loop TC-10 temperature controller (NPI) with a HPT-2 in-line heater (ALA). Temperature shifts from 25°C to 32°C were complete in <1 min.

### Structural Imaging

Fixed samples expressing fluorescent proteins and/or stained with fluorescently labeled antibodies were imaged on a Leica TCS SP5 confocal microscope (see the Supplemental Experimental Procedures).

## Supplemental Information

Document S1. Supplemental Experimental Procedures, Figures S1–S8, and Tables S1–S6

Movie S1. Dendritic and Axonal Arborizations of Neurons Labeled by the *Mz699-GAL4* Driver Line, Related to Figure 5 Stack of 67 confocal sections (1.5 μm) through the vlpr of a fly carrying *Mz699-GAL4:UAS-mCD8-GFP* transgenes. GFPDSyd-1 expression (cyan) labels presynaptic axonal terminals. DenMark expression (magenta) labels putative dendritic regions.

Movie S2. GABAergic iPNs, Related to Figure 5 Stack of 60 confocal sections (1.5 μm) through the antennal lobe of a fly carrying *Mz699-GAL4:UAS-mCD8-GFP* transgenes after immunostaining against GABA (magenta) and GFP (cyan) and counterstaining with the nuclear marker TOTO-3 (yellow).

Movie S3. vlpr Neurons, Related to Figure 5 Stack of 56 confocal sections (1.5 μm) through the vlpr of a fly carrying *Mz699-GAL4:UAS-mCD8-GFP* transgenes after immunostaining against GABA (magenta) and GFP (cyan) and counterstaining with the nuclear marker TOTO-3 (yellow).

## References

[bib1] Abbott L.F., Regehr W.G. (2004). Synaptic computation. Nature.

[bib2] Albus J.S. (1971). A theory of cerebellar function. Math. Biosci..

[bib3] Barlow H.B. (1953). Summation and inhibition in the frog’s retina. J. Physiol..

[bib4] Barlow H.B., Rosenblith W.A. (1961). Possible principles underlying the transformation of sensory messages. Sensory Communication.

[bib5] Claridge-Chang A., Roorda R.D., Vrontou E., Sjulson L., Li H., Hirsh J., Miesenböck G. (2009). Writing memories with light-addressable reinforcement circuitry. Cell.

[bib6] Clyne P.J., Warr C.G., Freeman M.R., Lessing D., Kim J., Carlson J.R. (1999). A novel family of divergent seven-transmembrane proteins: candidate odorant receptors in Drosophila. Neuron.

[bib7] Couto A., Alenius M., Dickson B.J. (2005). Molecular, anatomical, and functional organization of the Drosophila olfactory system. Curr. Biol..

[bib8] Dobritsa A.A., van der Goes van Naters W., Warr C.G., Steinbrecht R.A., Carlson J.R. (2003). Integrating the molecular and cellular basis of odor coding in the Drosophila antenna. Neuron.

[bib9] Fishilevich E., Vosshall L.B. (2005). Genetic and functional subdivision of the Drosophila antennal lobe. Curr. Biol..

[bib10] Gao Q., Yuan B., Chess A. (2000). Convergent projections of Drosophila olfactory neurons to specific glomeruli in the antennal lobe. Nat. Neurosci..

[bib11] Gold J.I., Shadlen M.N. (2001). Neural computations that underlie decisions about sensory stimuli. Trends Cogn. Sci..

[bib12] Good I.J., Bernardo J.M., DeGroot M.H., Lindley D.V., Smith A.F.M. (1985). Weight of evidence: A brief survey. Bayesian Statistics 2.

[bib13] Gray C.M., König P., Engel A.K., Singer W. (1989). Oscillatory responses in cat visual cortex exhibit inter-columnar synchronization which reflects global stimulus properties. Nature.

[bib14] Gupta N., Stopfer M. (2012). Functional analysis of a higher olfactory center, the lateral horn. J. Neurosci..

[bib15] Hallem E.A., Carlson J.R. (2006). Coding of odors by a receptor repertoire. Cell.

[bib16] Hallem E.A., Ho M.G., Carlson J.R. (2004). The molecular basis of odor coding in the Drosophila antenna. Cell.

[bib17] Hamada F.N., Rosenzweig M., Kang K., Pulver S.R., Ghezzi A., Jegla T.J., Garrity P.A. (2008). An internal thermal sensor controlling temperature preference in Drosophila. Nature.

[bib18] Hartline H.K., Wagner H.G., Ratliff F. (1956). Inhibition in the eye of Limulus. J. Gen. Physiol..

[bib19] Heimbeck G., Bugnon V., Gendre N., Keller A., Stocker R.F. (2001). A central neural circuit for experience-independent olfactory and courtship behavior in Drosophila melanogaster. Proc. Natl. Acad. Sci. USA.

[bib20] Heisenberg M. (2003). Mushroom body memoir: from maps to models. Nat. Rev. Neurosci..

[bib21] Heisenberg M., Borst A., Wagner S., Byers D. (1985). Drosophila mushroom body mutants are deficient in olfactory learning. J. Neurogenet..

[bib22] Jefferis G.S.X.E., Marin E.C., Stocker R.F., Luo L. (2001). Target neuron prespecification in the olfactory map of Drosophila. Nature.

[bib23] Jefferis G.S., Potter C.J., Chan A.M., Marin E.C., Rohlfing T., Maurer C.R.J., Luo L. (2007). Comprehensive maps of Drosophila higher olfactory centers: spatially segregated fruit and pheromone representation. Cell.

[bib24] Jortner R.A., Farivar S.S., Laurent G. (2007). A simple connectivity scheme for sparse coding in an olfactory system. J. Neurosci..

[bib25] Kazama H., Wilson R.I. (2008). Homeostatic matching and nonlinear amplification at identified central synapses. Neuron.

[bib26] Kitamoto T. (2001). Conditional modification of behavior in Drosophila by targeted expression of a temperature-sensitive shibire allele in defined neurons. J. Neurobiol..

[bib27] Knaden M., Strutz A., Ahsan J., Sachse S., Hansson B.S. (2012). Spatial representation of odorant valence in an insect brain. Cell Rep.

[bib28] Kreher S.A., Mathew D., Kim J., Carlson J.R. (2008). Translation of sensory input into behavioral output via an olfactory system. Neuron.

[bib29] Kuffler S.W. (1953). Discharge patterns and functional organization of mammalian retina. J. Neurophysiol..

[bib30] Kurtovic A., Widmer A., Dickson B.J. (2007). A single class of olfactory neurons mediates behavioural responses to a Drosophila sex pheromone. Nature.

[bib31] Lai S.L., Awasaki T., Ito K., Lee T. (2008). Clonal analysis of Drosophila antennal lobe neurons: diverse neuronal architectures in the lateral neuroblast lineage. Development.

[bib32] Laurent G., Davidowitz H. (1994). Encoding of olfactory information with oscillating neural assemblies. Science.

[bib33] Legenstein R., Maass W. (2008). On the classification capability of sign-constrained perceptrons. Neural Comput..

[bib34] Liang L., Li Y., Potter C.J., Yizhar O., Deisseroth K., Tsien R.W., Luo L. (2013). GABAergic Projection Neurons Route Selective Olfactory Inputs to Specific Higher-Order Neurons. Neuron.

[bib35] Luo S.X., Axel R., Abbott L.F. (2010). Generating sparse and selective third-order responses in the olfactory system of the fly. Proc. Natl. Acad. Sci. USA.

[bib36] Marr D. (1969). A theory of cerebellar cortex. J. Physiol..

[bib37] McGuire S.E., Le P.T., Osborn A.J., Matsumoto K., Davis R.L. (2003). Spatiotemporal rescue of memory dysfunction in Drosophila. Science.

[bib38] Miesenböck G., De Angelis D.A., Rothman J.E. (1998). Visualizing secretion and synaptic transmission with pH-sensitive green fluorescent proteins. Nature.

[bib39] Mysore S.P., Knudsen E.I. (2012). Reciprocal inhibition of inhibition: a circuit motif for flexible categorization in stimulus selection. Neuron.

[bib40] Ng M., Roorda R.D., Lima S.Q., Zemelman B.V., Morcillo P., Miesenböck G. (2002). Transmission of olfactory information between three populations of neurons in the antennal lobe of the fly. Neuron.

[bib41] Okada R., Awasaki T., Ito K. (2009). Gamma-aminobutyric acid (GABA)-mediated neural connections in the Drosophila antennal lobe. J. Comp. Neurol..

[bib42] Olsen S.R., Wilson R.I. (2008). Lateral presynaptic inhibition mediates gain control in an olfactory circuit. Nature.

[bib43] Olsen S.R., Bhandawat V., Wilson R.I. (2010). Divisive normalization in olfactory population codes. Neuron.

[bib44] Root C.M., Masuyama K., Green D.S., Enell L.E., Nässel D.R., Lee C.H., Wang J.W. (2008). A presynaptic gain control mechanism fine-tunes olfactory behavior. Neuron.

[bib45] Semmelhack J.L., Wang J.W. (2009). Select Drosophila glomeruli mediate innate olfactory attraction and aversion. Nature.

[bib46] Stensmyr M.C., Dweck H.K.M., Farhan A., Ibba I., Strutz A., Mukunda L., Linz J., Grabe V., Steck K., Lavista-Llanos S. (2012). A conserved dedicated olfactory circuit for detecting harmful microbes in Drosophila. Cell.

[bib47] Stocker R.F., Lienhard M.C., Borst A., Fischbach K.F. (1990). Neuronal architecture of the antennal lobe in Drosophila melanogaster. Cell Tissue Res..

[bib48] Stopfer M., Bhagavan S., Smith B.H., Laurent G. (1997). Impaired odour discrimination on desynchronization of odour-encoding neural assemblies. Nature.

[bib49] Suh G.S., Wong A.M., Hergarden A.C., Wang J.W., Simon A.F., Benzer S., Axel R., Anderson D.J. (2004). A single population of olfactory sensory neurons mediates an innate avoidance behaviour in Drosophila. Nature.

[bib50] Tanaka N.K., Endo K., Ito K. (2012). Organization of antennal lobe-associated neurons in adult Drosophila melanogaster brain. J. Comp. Neurol..

[bib51] Tian L., Hires S.A., Mao T., Huber D., Chiappe M.E., Chalasani S.H., Petreanu L., Akerboom J., McKinney S.A., Schreiter E.R. (2009). Imaging neural activity in worms, flies and mice with improved GCaMP calcium indicators. Nat. Methods.

[bib52] van der Goes van Naters W., Carlson J.R. (2007). Receptors and neurons for fly odors in Drosophila. Curr. Biol..

[bib53] Vosshall L.B., Amrein H., Morozov P.S., Rzhetsky A., Axel R. (1999). A spatial map of olfactory receptor expression in the Drosophila antenna. Cell.

[bib54] Vosshall L.B., Wong A.M., Axel R. (2000). An olfactory sensory map in the fly brain. Cell.

[bib55] Wang J.W., Wong A.M., Flores J., Vosshall L.B., Axel R. (2003). Two-photon calcium imaging reveals an odor-evoked map of activity in the fly brain. Cell.

[bib56] Wilson R.I., Laurent G. (2005). Role of GABAergic inhibition in shaping odor-evoked spatiotemporal patterns in the Drosophila antennal lobe. J. Neurosci..

