# Supplementary material for: Odor Discrimination in Drosophila: From Neural Population Codes to Behavior
Source: Neuron. 2013 Sep 4;79(5):932–44. doi: 10.1016/j.neuron.2013.08.006 (PMC3765961; doi:10.1016/j.neuron.2013.08.006)
Supplement: Document S1. Supplemental Experimental Procedures, Figures S1–S8, and Tables S1–S6 [file mmc1.pdf]

**Neuron, Volume *79***
**Supplemental Information**

# Odor Discrimination in Drosophila: From Neural Population Codes to Behavior

**Moshe Parnas, Andrew C. Lin, Wolf Huetteroth, and Gero Miesenböck**

## Supplemental Material

### Supplemental Figures and Legends

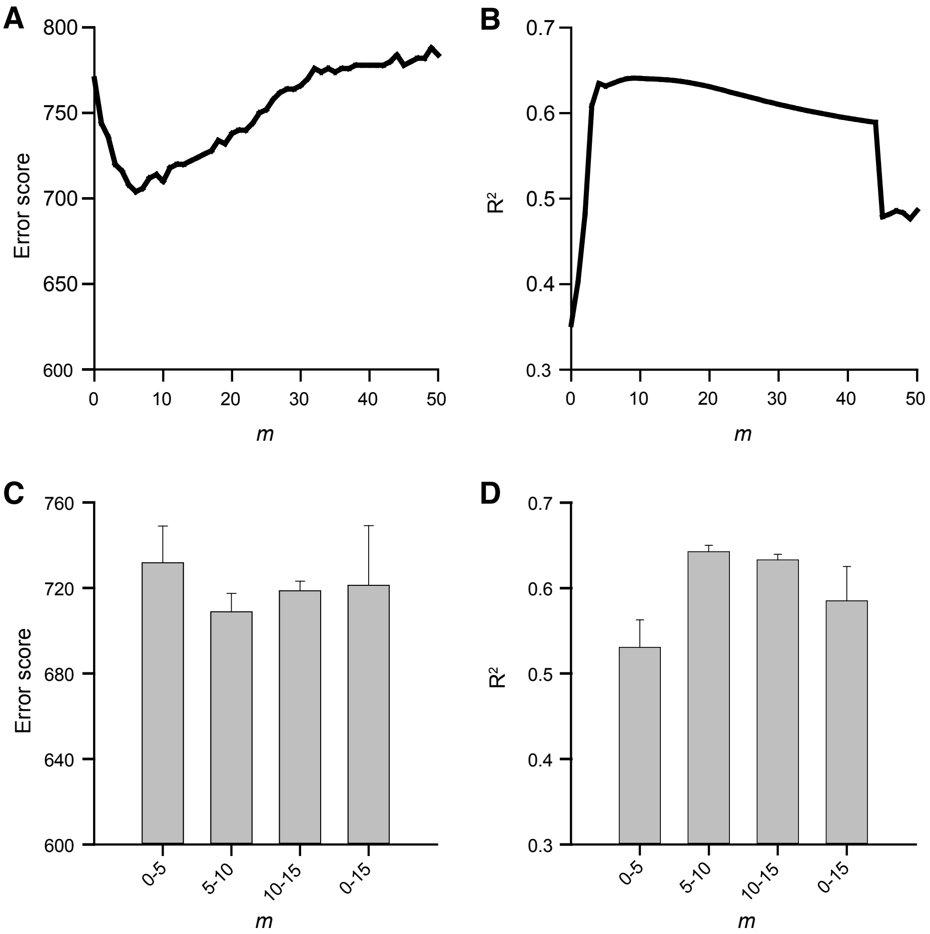


**Figure S1, Related to Figure 1. Sensitivity Analysis for Computing ePN Activity Vectors**
ePN activity vectors were computed from ORN activity vectors using a two-parameter transformation (Olsen et al., 2010; see equation in the legend to Figure 1). Sensitivity analysis was performed to evaluate how the choice of a particular numerical value for *m* would impact the accuracy with which odor discrimination could be predicted from distances between ePN activity vectors (Figure 2D).

(A) Prediction error scores as a function of *m*. ePN activity vectors were computed for values of *m* ranging from 0 to 50. The decision bias scores elicited by 51 odor pairs (Figure 2D) were rank-ordered and compared to the rank-ordered Euclidean distances between the corresponding ePN

activity vectors. Each mismatch between the two rank-ordered lists attracted a penalty score. For example, an odor pairing that ranked third in decision bias but sixth in ePN distance received three penalty points. The penalty points of all odor combinations were summed into a combined error score, which is displayed. The error score is minimal between *m* = 5 and *m* = 12.
(B) $R^2$ scores as a function of *m*. ePN activity vectors were computed for values of *m* ranging from 0 to 50. The decision bias scores elicited by 51 odor pairs (Figure 2D) were fitted by logistic functions of the Euclidean distance between ePN activity vectors. The effective $R^2$ score peaks at *m* ~10.
(C) Prediction error scores as a function of interglomerular variation of *m*. A different value of *m*, in the indicated ranges, was chosen randomly for each of the 24 glomeruli with characterized ORN response spectra (Hallem and Carlson, 2006; Hallem et al., 2004), and error scores (mean ± SD, n = 1,000 iterations) were computed as in panel A. The error scores are minimal between *m* = 5 and *m* = 15.
(D) $R^2$ scores as a function of interglomerular variation of *m*. A different value of *m*, in the indicated range, was chosen randomly for each of the 24 glomeruli with characterized ORN response spectra (Hallem and Carlson, 2006; Hallem et al., 2004), and $R^2$ scores (mean ± SD, n = 1,000 iterations) were computed as in panel B. The $R^2$ scores peak between *m* = 5 and *m* = 15.

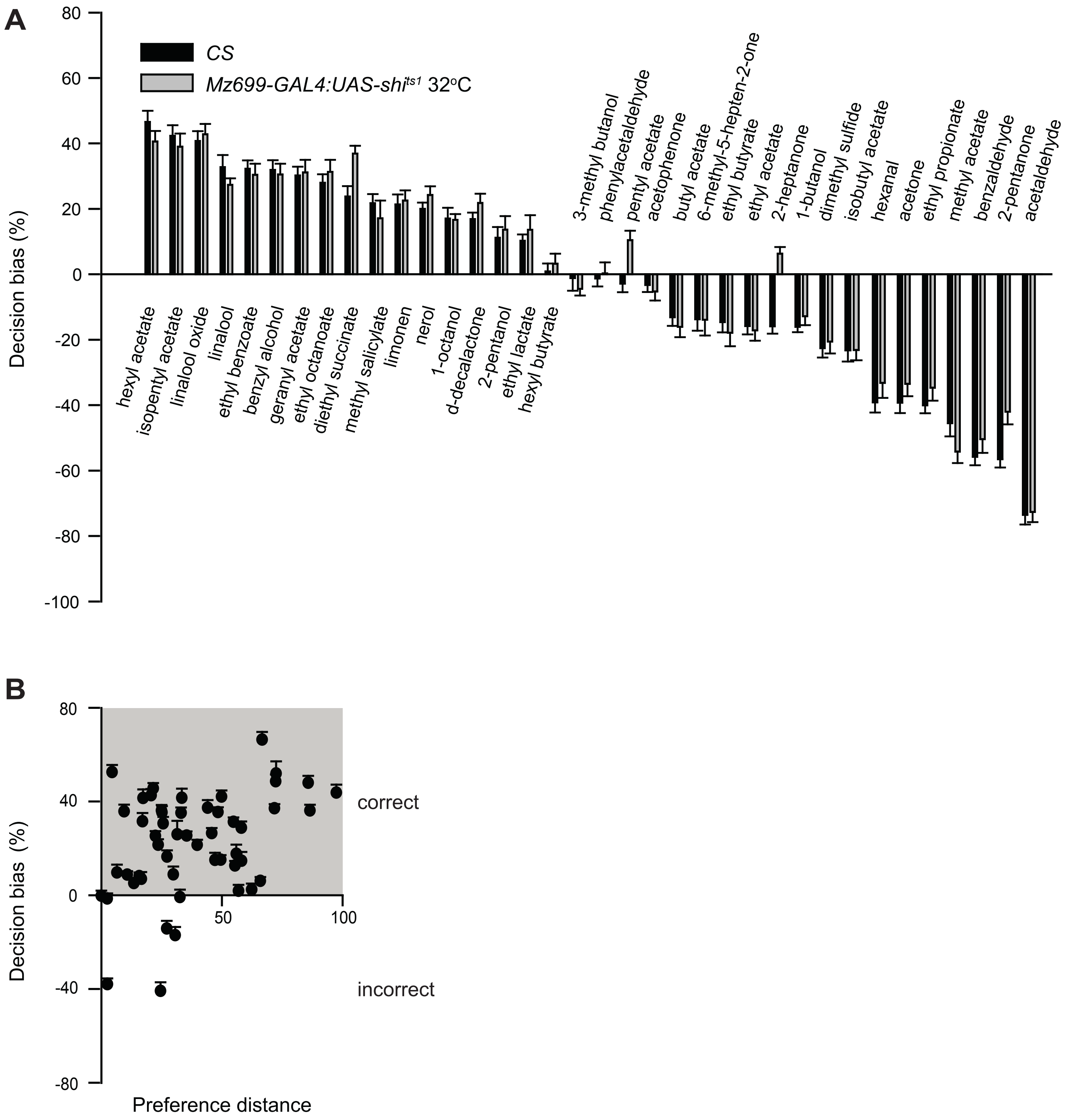


**Figure S2, Related to Figure 2. Decision Bias in Odor-Air and Odor-Odor Trials**

(A) Odor valence. The hedonic valences of the individual odors used in Figure 2 were determined against air washed through mineral oil (mean ± SEM, n = 40–120 flies per odor). Positive decision bias scores indicate attraction; negative decision bias scores indicate

avoidance. Black columns denote the preferences of Canton-S flies at 32 ˚C; gray columns denote the preferences of flies carrying *Mz699-GAL4:UAS-shi*$^{ts1}$ transgenes at the restrictive temperature of 32 ˚C, when iPN output is blocked. See also Figure 6D.

(B) Decision bias scores elicited by 51 odor pairs as a function of preference distance (mean ± SEM, n = 40–120 flies per data point, $R^2$ = 0.14). Pairwise preference distances for each pair of odors A and B were generated by subtracting the hedonic valences obtained in panel A: preference distance = (preference for odor A over air) – (preference for odor B over air). Because the assignment of odors in each pair to A or B is arbitrary, we assigned them such that preference distances would be positive. Sign information was preserved by matching the A vs. B labeling in the decision bias scores to that in the preference distances. In most cases the preference distance correctly predicts the sign of the decision bias (shaded area).

See also Table S2.

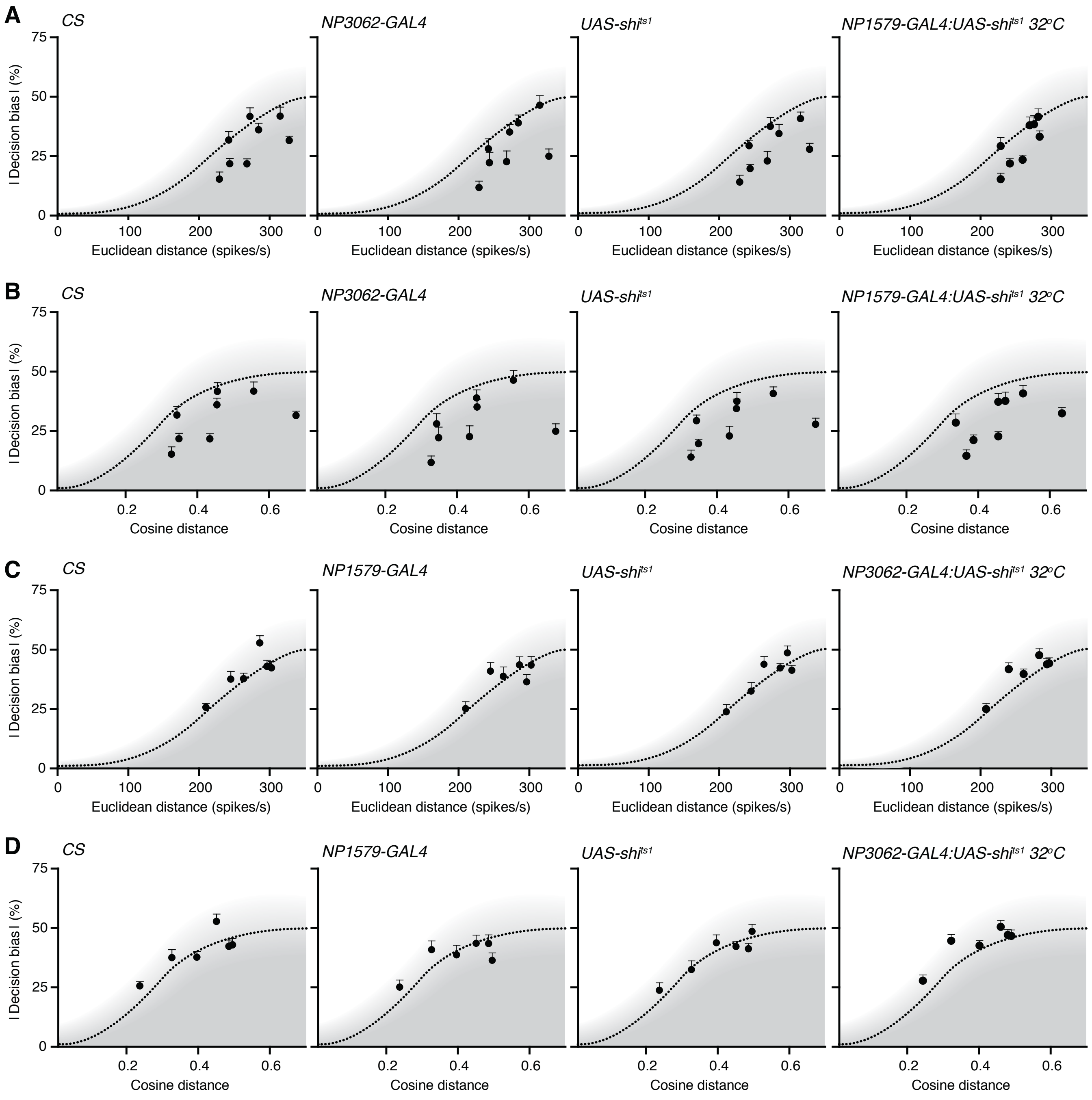


**Figure S3, Related to Figure 3. Control Data for Experimental Manipulations of Distance-Based Discrimination**

(A, B) Absolute decision bias scores of control strains used in Figure 3C as functions of Euclidean (A) or cosine distances (B) between ePN activity vectors (mean ± SEM, n = 40–80 flies per data point). The distance-discrimination functions obtained in Figure 2D are reproduced for

reference. Blocking output of *NP1579-GAL4*-positive neurons does not affect performance for odor pairs whose distances should be specifically impacted by blocking output of *NP3062-GAL4*-positive neurons (right panels).
(C, D) Absolute decision bias scores of control strains used in Figure 3F as functions of Euclidean (C) or cosine distances (D) between ePN activity vectors (mean ± SEM, n = 40–80 flies per data point). The distance-discrimination functions obtained in Figure 2D are reproduced for reference. Blocking output of *NP3062-GAL4*-positive neurons does not affect performance for odor pairs whose distances should be specifically impacted by blocking output of *NP1579-GAL4*-positive neurons (right panels).
See also Table S3.

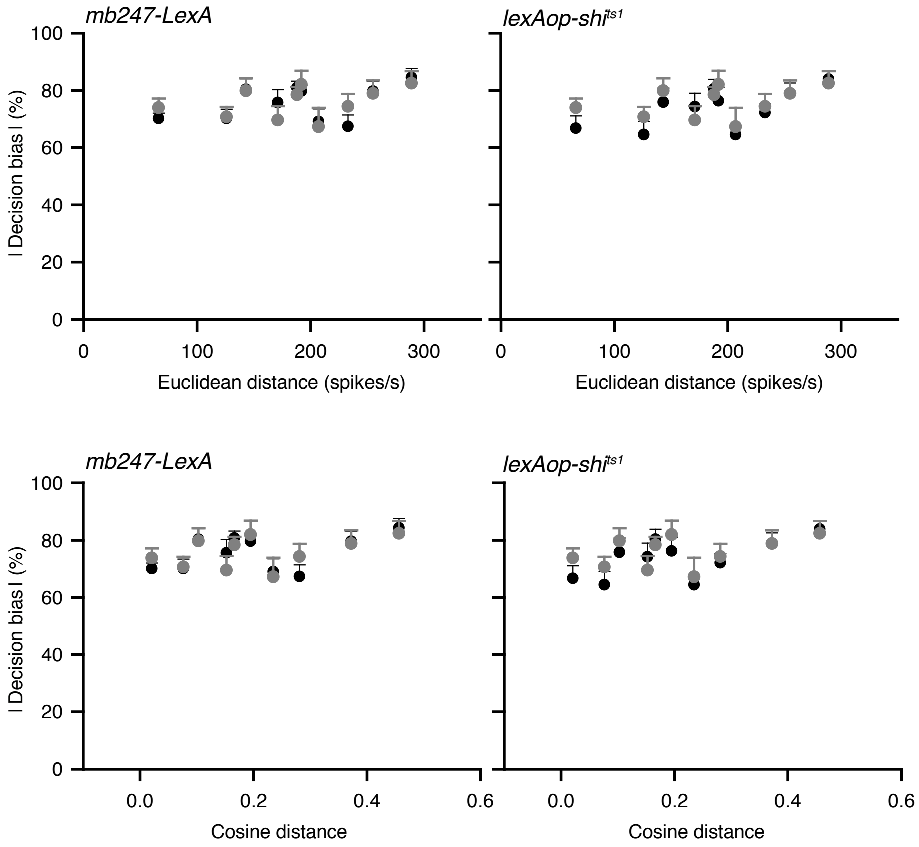


**Figure S4, Related to Figure 4. Control Data for the Analysis of Innate vs. Trained Discrimination**

Absolute decision bias scores of control strains used in Figure 4 as functions of Euclidean or cosine distances between ePN activity vectors (black; mean ± SEM, n = 30–60 flies per data point). The decision bias scores of wild-type (CS) flies are shown for reference (gray; mean ± SEM, n = 30–60 flies per data point).

See also Table S4.

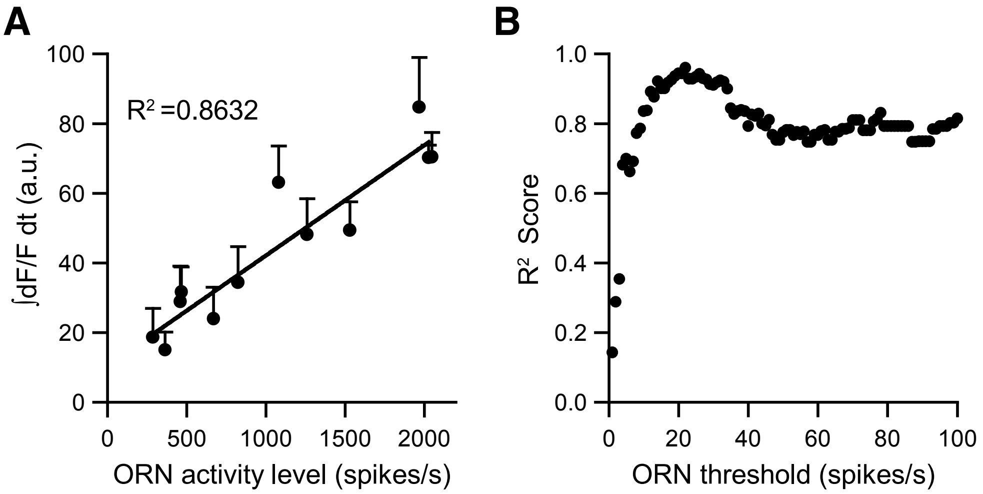


**Figure S5, Related to Figure 5. iPN Activity Scales with Excitation in the Antennal Lobe**
(A) Integrated GCaMP3 fluorescence transients (area under the fluorescence trace during a 5 s odor pulse) in iPN axons as a function of the cumulative ORN spike rate an odor elicits (mean ± SEM, $p < 0.0001$, $n = 11$ flies per data point).
(B) iPN output scales with the number of active glomerular channels. ORN activity vectors were thresholded at spike rates ranging from 0 to 100 spikes/s. A glomerulus was considered active if the spike rate of its cognate ORNs exceeded the threshold. For each threshold level, the number of active glomeruli active was correlated with the integrated fluorescence transient (area under the fluorescence trace during a 5 s odor pulse) in iPN axons. The $R^2$ score of the correlation peaks at a threshold value of 20–33 spikes/s.

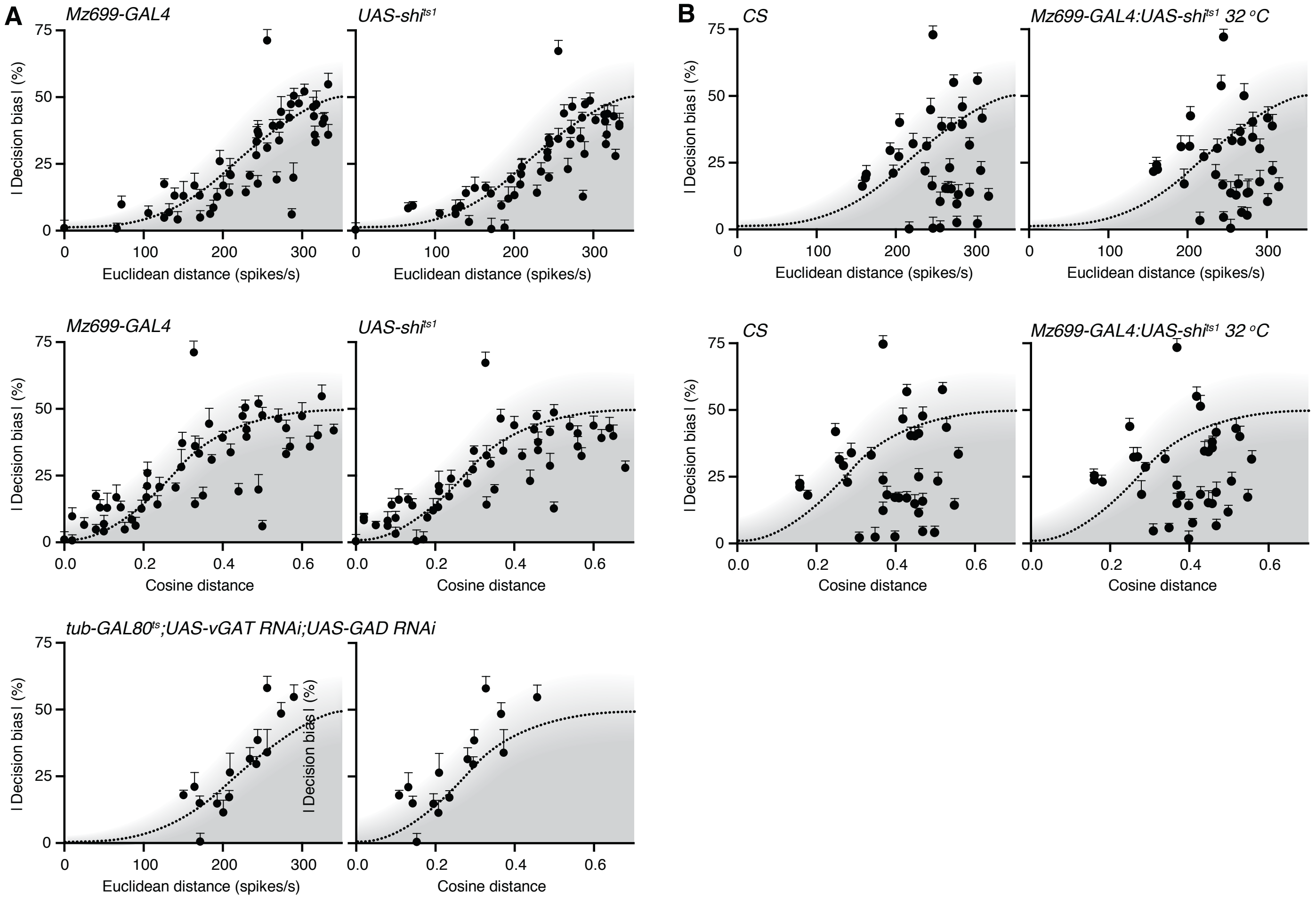


**Figure S6, Related to Figure 6. Control Data for the Analysis of the Role of iPN Inhibition in Odor Discrimination**

(A) Absolute decision bias scores of control strains used in Figures 6A–6C as functions of Euclidean or cosine distances between ePN activity vectors (mean ± SEM, n = 40–60 flies per data point). The distance-discrimination functions obtained in Figure 2D are reproduced for reference.

See also Tables S5 and S6.

(B) Absolute decision bias scores elicited by 36 odors vs. air as functions of Euclidean and cosine distances between ePN activity vectors (mean ± SEM, n = 40–80 flies per data point). ePN activity vectors in air were calculated from measured spontaneous ORN activity (Hallem and Carlson, 2006; Hallem et al., 2004). See also Table S2 and Figure S2.

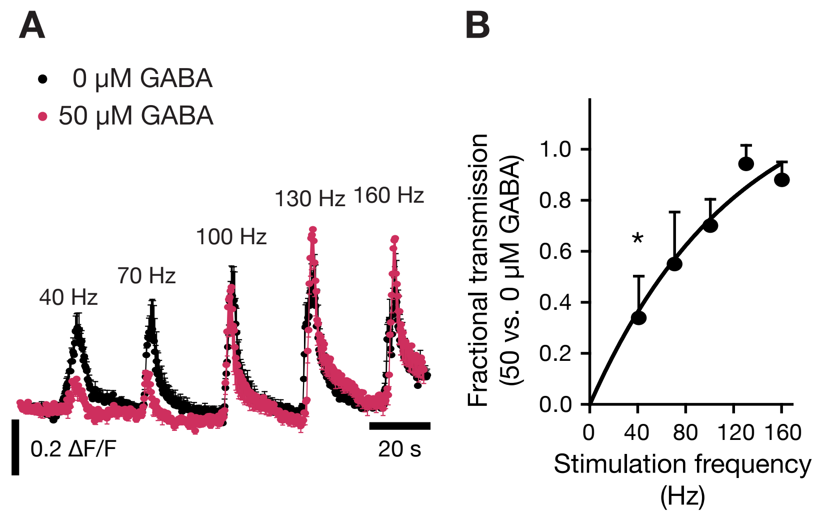


**Figure S7, Related to Figure 7. iPN Inhibition Imposes a High-Pass Filter on ePN Synaptic Output**

(A) Average spH fluoresence changes in ePN projections to the LH, evoked by a total of 200 electrical stimulation pulses at the indicated frequencies, in the absence (black) or presence (red) of 50 μM GABA (mean ± SEM, n = 4 flies).

(B) Average ratio of integrated spH fluorescence transients (areas under the fluorescence trances during electrical stimulation) in the presence and absence of 50 μM GABA (mean ± SEM, n = 4 flies). The ratios of ΔF/F with 0 vs. 50 μm GABA differ across frequencies ($p < 0.0001$; one-way repeated-measures ANOVA). The asterisk indicates a significant difference between the presence and absence of 50 μM GABA (*$p < 0.05$, paired t-test).

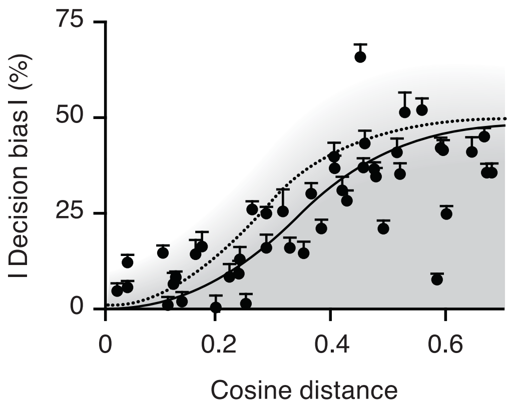


**Figure S8, Related to Figure 8. The Distance-Discrimination Function after Correction for the Effect of the Inhibitory High-Pass Filter**

Absolute decision bias scores elicited by 50 odor pairs (see Figure 2D) as a function of cosine distances after computational correction for the effect of the inhibitory high-pass filter. The corrected distance-discrimination function (solid line) was obtained from a least-squares logistic fit to the data; the fit was constrained to include the origin ($R^2$= 0.6448, $p < 0.0001$). The distance-discrimination function obtained in Figure 2D is reproduced for reference (dotted line).

## SUPPLEMENTAL TABLES

## Table S1, Related to Figure 2. Decision Bias Scores in Odor-Odor Trials

| | Odors | | Euclidean distance | Cosine distance | Decision bias (%) |
|---|---|---|---|---|---|
| | Odor A | Odor B | CS | CS | CS |
| **1** | 1-Octanol | 1-Octanol | 0 | 0 | 0.0 ± 2.8 |
| **2** | 2-Heptanone | Pentyl acetate | 66 | 0.02 | -5.4 ± 2.1 |
| **3** | Butyl acetate | Isopentyl acetate | 72 | 0.02 | -12.8 ± 2.0 |
| **4** | Isopentyl acetate | Isobutyl acetate | 107 | 0.05 | 6.3 ± 1.7 |
| **5** | 2-Heptanone | Butyl acetate | 126 | 0.08 | 1.7 ± 2.2 |
| **6** | Hexyl acetate | Pentyl acetate | 126 | 0.08 | 15.3 ± 2.0 |
| **7** | 1-Octanol | Hexyl butyrate | 132 | 0.10 | 7.2 ± 2.9 |
| **8** | Butyl acetate | Pentyl acetate | 139 | 0.09 | -9.0 ± 1.5 |
| **9** | Hexyl acetate | 2-Heptanone | 143 | 0.10 | 2.6 ± 2.5 |
| **10** | Isopentyl acetate | 2-Heptanone | 150 | 0.11 | 14.9 ± 3.8 |
| **11** | Isopentyl acetate | 6-Methyl-5-hepten-2-one | 164 | 0.13 | 16.9 ± 3.9 |
| **12** | 1-Octanol | 2-Heptanone | 171 | 0.15 | -1.1 ± 3.2 |
| **13** | 2-Pentanol | 2-Heptanone | 171 | 0.14 | -13.6 ± 3.3 |
| **14** | 1-Octanol | Hexyl acetate | 184 | 0.18 | -9.1 ± 3.4 |
| **15** | Isopentyl acetate | Ethyl butyrate | 188 | 0.17 | 2.1 ± 2.6 |
| **16** | 1-Octanol | 2-Pentanol | 192 | 0.19 | 9.9 ± 3.4 |
| **17** | Hexyl acetate | Hexyl butyrate | 196 | 0.21 | 26.7 ± 2.2 |
| **18** | 2-Pentanol | Ethyl acetate | 200 | 0.21 | 16.7 ± 2.7 |
| **19** | 1-Octanol | 6-Methyl-5-hepten-2-one | 207 | 0.24 | -16.7 ± 3.5 |
| **20** | Isopentyl acetate | 2-Pentanol | 208 | 0.21 | 26.2 ± 5.8 |
| **21** | Acetophenone | Benzyl alcohol | 210 | 0.24 | -25.7 ± 1.8 |
| **22** | Dimethyl sulfide | Ethyl octanoate | 229 | 0.33 | -15.2 ± 3.1 |
| **23** | 1-Octanol | Isopentyl acetate | 233 | 0.28 | -30.9 ± 2.8 |
| **24** | Isopentyl acetate | Ethyl acetate | 242 | 0.30 | 29.4 ± 2.7 |
| **25** | Dimethyl sulfide | Hexanal | 242 | 0.34 | 31.7 ± 3.6 |
| **26** | 2-Pentanol | 6-Methyl-5-hepten-2-one | 244 | 0.30 | -40.5 ± 3.7 |
| **27** | Dimethyl sulfide | Nerol | 244 | 0.35 | -21.7 ± 2.4 |
| **28** | Acetophenone | Linalool oxide | 245 | 0.33 | -37.5 ± 3.3 |
| **29** | 1-Octanol | Ethyl acetate | 255 | 0.37 | 35.3 ± 2.3 |
| **30** | 2-Pentanol | Benzaldehyde | 256 | 0.33 | 66.6 ± 3.3 |
| **31** | Acetophenone | Diethyl succinate | 263 | 0.40 | -37.7 ± 2.5 |
| **32** | Dimethyl sulfide | 1-Octanol | 268 | 0.44 | -21.7 ± 2.2 |
| **33** | Linalool | Acetone | 271 | 0.42 | 37.3 ± 1.8 |
| **34** | Dimethyl sulfide | Diethyl succinate | 272 | 0.46 | -41.7 ± 3.7 |
| **35** | Isopentyl acetate | Benzaldehyde | 273 | 0.37 | 44.5 ± 3.4 |
| **36** | Dimethyl sulfide | 6-Methyl-5-hepten-2-one | 284 | 0.46 | -36.0 ± 2.9 |
| **37** | Acetophenone | Nerol | 286 | 0.45 | -52.7 ± 3.1 |
| **38** | Ethyl benzoate | 1-Octanol | 287 | 0.50 | 8.3 ± 1.6 |
| **39** | 1-Octanol | Benzaldehyde | 289 | 0.46 | 52.1 ± 5.2 |
| **40** | Ethyl benzoate | Ethyl lactate | 289 | 0.49 | 25.5 ± 2.1 |
| **41** | Acetophenone | 1-Octanol | 296 | 0.50 | -42.8 ± 2.7 |
| **42** | Acetophenone | Hexyl acetate | 303 | 0.49 | -42.3 ± 2.7 |
| **43** | Ethyl benzoate | 2-Pentanol | 314 | 0.54 | 45.8 ± 2.3 |
| **44** | Dimethyl sulfide | Benzaldehyde | 315 | 0.56 | 41.8 ± 3.9 |
| **45** | Geranyl acetate | 2-Pentanone | 316 | 0.57 | 36.3 ± 2.5 |
| **46** | Pentyl acetate | Methyl salicylate | 317 | 0.56 | 36.4 ± 2.4 |
| **47** | δ-Decalactone | Benzaldehyde | 318 | 0.60 | 48.8 ± 2.9 |
| **48** | Hexyl acetate | Methyl salicylate | 326 | 0.64 | 35.6 ± 2.7 |
| **49** | Dimethyl sulfide | Ethyl benzoate | 328 | 0.68 | -31.5 ± 1.9 |
| **50** | Geranyl acetate | Benzaldehyde | 333 | 0.65 | 48.2 ± 3.0 |
| **51** | 1-Butanol | Ethyl benzoate | 333 | 0.62 | -35.7 ± 2.1 |

Decision bias scores of Canton-S flies in all odor-odor trials shown in Figure 2D. Odor pairs are sorted according to the Euclidean distance between the predicted ePN activity vectors of the two odors. A positive decision bias score indicates a preference for odor A; a negative decision bias score indicates a preference for odor B. Scores are given as mean ± SEM (n = 40–80 flies per data point)

**Table S2, Related to Figures 2, 6, and S2. Decision Bias Scores of CS and *Mz699-GAL4:UAS-shi*$^{ts1}$ Flies in Odor-Air Trials**

| | Odors | | Euclidean distance | Cosine distance | Decision bias (%) | |
|---|---|---|---|---|---|---|
| | Odor | Air | CS | CS | CS | *Mz699-GAL4:UAS-shi*$^{ts1}$ at 32$^{0}$C |
| 1 | δ-Decalactone | Mineral oil | 158 | 0.18 | 16.9 ± 2.0 | 21.8 ± 2.8 |
| 2 | Nerol | Mineral oil | 162 | 0.16 | 20.0 ± 1.9 | 24.2 ± 2.7 |
| 3 | Limonen | Mineral oil | 163 | 0.16 | 21.3 ± 3.1 | 22.6 ± 3.1 |
| 4 | Geranyl acetate | Mineral oil | 193 | 0.26 | 30.3 ± 2.6 | 31.1 ± 3.9 |
| 5 | Methyl salicylate | Mineral oil | 197 | 0.28 | 21.8 ± 2.8 | 17.1 ± 5.4 |
| 6 | Ethyl octanoate | Mineral oil | 204 | 0.27 | 28.0 ± 2.6 | 31.3 ± 3.7 |
| 7 | Linalool oxide | Mineral oil | 205 | 0.25 | 40.8 ± 3.0 | 42.8 ± 3.2 |
| 8 | Hexyl butyrate | Mineral oil | 217 | 0.31 | 0.9 ± 2.4 | 3.3 ± 3.1 |
| 9 | Linalool | Mineral oil | 222 | 0.29 | 32.8 ± 3.7 | 27.4 ± 2.0 |
| 10 | Dimethyl sulfide | Mineral oil | 237 | 0.37 | -22.6 ± 2.8 | -20.6 ± 3.6 |
| 11 | Benzyl alcohol | Mineral oil | 239 | 0.34 | 31.9 ± 2.9 | 30.5 ± 3.3 |
| 12 | Methyl acetate | Mineral oil | 244 | 0.42 | -45.5 ± 4.0 | -54.2 ± 3.6 |
| 13 | 1-Octanol | Mineral oil | 246 | 0.38 | 17.0 ± 3.3 | 16.7 ± 1.7 |
| 14 | 3-Methylbutanol | Mineral oil | 247 | 0.35 | -1.2 ± 3.3 | -4.4 ± 2.0 |
| 15 | Acetaldehyde | Mineral oil | 247 | 0.37 | -73.6 ± 2.9 | -72.6 ± 3.2 |
| 16 | 2-Pentanol | Mineral oil | 256 | 0.37 | 11.1 ± 3.3 | 13.7 ± 4.1 |
| 17 | Phenylacetaldehyde | Mineral oil | 256 | 0.40 | -1.3 ± 2.4 | 0.3 ± 3.3 |
| 18 | Acetone | Mineral oil | 258 | 0.44 | -39.3 ± 3.1 | -33.5 ± 3.8 |
| 19 | 1-Butanol | Mineral oil | 263 | 0.40 | -16.1 ± 1.6 | -12.8 ± 2.7 |
| 20 | Ethyl acetate | Mineral oil | 266 | 0.43 | -15.8 ± 2.6 | -17.1 ± 3.2 |
| 21 | Diethyl succinate | Mineral oil | 268 | 0.46 | 23.8 ± 3.2 | 36.9 ± 2.4 |
| 22 | 2-Heptanone | Mineral oil | 270 | 0.41 | -15.9 ± 2.2 | 6.3 ± 2.0 |
| 23 | Hexanal | Mineral oil | 270 | 0.45 | -39.2 ± 3.2 | -33.2 ± 4.6 |
| 24 | Benzaldehyde | Mineral oil | 273 | 0.43 | -55.8 ± 2.6 | -50.4 ± 4.6 |
| 25 | Ethyl lactate | Mineral oil | 277 | 0.46 | 10.2 ± 1.9 | 13.6 ± 4.5 |
| 26 | Acetophenone | Mineral oil | 277 | 0.47 | -3.3 ± 2.2 | -5.2 ± 2.8 |
| 27 | 6-Methyl-5-hepten-2-one | Mineral oil | 279 | 0.45 | -13.7 ± 3.5 | -13.9 ± 4.8 |
| 28 | Hexyl acetate | Mineral oil | 284 | 0.47 | 46.6 ± 3.3 | 40.6 ± 3.2 |
| 29 | Ethyl propionate | Mineral oil | 284 | 0.46 | -40.0 ± 2.4 | -34.7 ± 3.9 |
| 30 | Ethyl benzoate | Mineral oil | 293 | 0.56 | 32.3 ± 2.5 | 30.4 ± 3.4 |
| 31 | Ethyl butyrate | Mineral oil | 293 | 0.47 | -14.6 ± 3.1 | -17.9 ± 4.1 |
| 32 | Pentyl acetate | Mineral oil | 303 | 0.50 | -2.9 ± 2.6 | 10.4 ± 2.9 |
| 33 | 2-Pentanone | Mineral oil | 303 | 0.52 | -56.5 ± 2.6 | -42.0 ± 3.9 |
| 34 | Isobutyl acetate | Mineral oil | 306 | 0.52 | -23.8 ± 3.5 | -23.7 ± 3.2 |
| 35 | Isopentyl acetate | Mineral oil | 309 | 0.53 | 42.3 ± 3.2 | 38.0 ± 4.0 |
| 36 | Butyl acetate | Mineral oil | 317 | 0.55 | -13.1 ± 2.6 | -16.0 ± 3.2 |

Decision bias scores against air of all odors used in this study. Odors were tested individually against air washed through mineral oil and are sorted according to the Euclidean distance between the predicted ePN activity vector of the odor and the predicted spontaneous ePN activity vector. A positive decision bias score indicates a preference for the odor; a negative decision bias score indicates a preference against the odor. Scores are given as mean ± SEM (n = 40–120 flies per data point).

**Table S3, Related to Figures 3 and S3. Decision Bias Scores of *NP3062-GAL4:UAS-shi$^{ts1}$* Flies , *NP1579-GAL4:UAS-shi$^{ts1}$* Flies, and Parental Control Strains**

| | Odors | | Euclidean distance | | | Cosine distance | | | Decision bias (%) | | | | |
|---|---|---|---|---|---|---|---|---|---|---|---|---|---|
| | Odor A | Odor B | CS | *NP3062-GAL4: UAS-shi$^{ts1}$* 32$^0$C | *NP1579-GAL4: UAS-shi$^{ts1}$* 32$^0$C | CS | *NP3062-GAL4: UAS-shi$^{ts1}$* 32$^0$C | *NP1579-GAL4: UAS-shi$^{ts1}$* 32$^0$C | *NP3062-GAL4: UAS-shi$^{ts1}$* 32$^0$C | *NP1579-GAL4: UAS-shi$^{ts1}$* 32$^0$C | *NP3062-GAL4: UAS-shi$^{ts1}$* 25$^0$C | *NP3062-GAL4* | *UAS-shi$^{ts1}$* |
| 1 | Dimethyl sulfide | Ethyl octanoate | 229 | 186 | 229 | 0.33 | 0.24 | 0.36 | -1.3 ± 2.5 | -14.4 ± 2.6 | -15.9 ± 2.4 | -11.8 ± 2.8 | -14.1 ± 3.0 |
| 2 | Dimethyl sulfide | Hexanal | 242 | 186 | 229 | 0.34 | 0.26 | 0.33 | 10.9 ± 5.0 | 28.4 ± 3.7 | 30.1 ± 5.3 | 28.0 ± 4.3 | 29.4 ± 2.4 |
| 3 | Dimethyl sulfide | Nerol | 244 | 201 | 242 | 0.35 | 0.26 | 0.38 | -9.5 ± 2.6 | -21.1 ± 2.2 | -18.0 ± 2.7 | -22.2 ± 4.5 | -19.7 ± 1.9 |
| 4 | Dimethyl sulfide | 1-Octanol | 268 | 231 | 260 | 0.44 | 0.36 | 0.45 | -21.0 ± 3.7 | -22.6 ± 2.0 | -25.4 ± 4.0 | -22.6 ± 4.6 | -22.9 ± 4.1 |
| 5 | Dimethyl sulfide | Diethyl succinate | 272 | 237 | 270 | 0.46 | 0.38 | 0.51 | -20.0 ± 2.8 | -37.2 ± 3.5 | -42.0 ± 3.1 | -35.1 ± 3.3 | -37.4 ± 3.8 |
| 6 | Dimethyl sulfide | 6-Methyl-5-hepten-2-one | 284 | 250 | 276 | 0.46 | 0.38 | 0.47 | -32.3 ± 3.8 | -37.6 ± 3.7 | -31.6 ± 2.5 | -38.9 ± 3.5 | -34.4 ± 4.0 |
| 7 | Dimethyl sulfide | Benzaldehyde | 315 | 267 | 282 | 0.56 | 0.50 | 0.52 | 43.1 ± 3.3 | 40.7 ± 3.4 | 44.5 ± 5.5 | 46.4 ± 4.0 | 40.7 ± 2.8 |
| 8 | Dimethyl sulfide | Ethyl benzoate | 328 | 299 | 284 | 0.68 | 0.63 | 0.63 | -36.2 ± 4.7 | -32.3 ± 2.5 | -27.3 ± 2.2 | -24.9 ± 3.1 | -27.8 ± 2.6 |
| | Odor A | Odor B | CS | *NP3062-GAL4: UAS-shi$^{ts1}$* 32$^0$C | *NP1579-GAL4: UAS-shi$^{ts1}$* 32$^0$C | CS | *NP3062-GAL4: UAS-shi$^{ts1}$* 32$^0$C | *NP1579-GAL4: UAS-shi$^{ts1}$* 32$^0$C | *NP3062-GAL4: UAS-shi$^{ts1}$* 32$^0$C | *NP1579-GAL4: UAS-shi$^{ts1}$* 32$^0$C | *NP1579-GAL4: UAS-shi$^{ts1}$* 25$^0$C | *NP1579-GAL4* | *UAS-shi$^{ts1}$* |
| 1 | Acetophenone | Benzyl alcohol | 210 | 209 | 143 | 0.24 | 0.24 | 0.12 | -25.1 ± 2.4 | -17.8 ± 2.2 | -23.9 ± 2.9 | -25.1 ± 3.0 | -23.7 ± 3.2 |
| 2 | Acetophenone | Linalool oxide | 245 | 241 | 185 | 0.33 | 0.32 | 0.23 | -41.9 ± 2.7 | -21.8 ± 2.1 | -34.3 ± 2.7 | -40.8 ± 3.7 | -32.5 ± 3.7 |
| 3 | Acetophenone | Diethyl succinate | 263 | 262 | 197 | 0.40 | 0.40 | 0.27 | -39.9 ± 2.1 | -26.1 ± 2.0 | -43.5 ± 2.2 | -38.7 ± 4.1 | -43.8 ± 3.4 |
| 4 | Acetophenone | Nerol | 286 | 284 | 233 | 0.45 | 0.46 | 0.36 | -47.8 ± 2.7 | -40.4 ± 2.9 | -36.9 ± 2.8 | -43.6 ± 3.4 | -42.2 ± 2.1 |
| 5 | Acetophenone | 1-Octanol | 296 | 295 | 244 | 0.50 | 0.49 | 0.40 | -44.0 ± 2.5 | -41.9 ± 2.9 | -40.5 ± 2.5 | -36.4 ± 3.2 | -48.6 ± 3.0 |
| 6 | Acetophenone | Hexyl acetate | 303 | 298 | 257 | 0.49 | 0.48 | 0.40 | -44.4 ± 2.3 | -40.3 ± 3.3 | -40.8 ± 2.4 | -43.4 ± 3.7 | -41.2 ± 2.2 |

Decision bias scores of all odor pairs shown in Figures 3 and S3. Odor pairs are sorted according to the Euclidean distance between the predicted ePN activity vectors of the two odors. A positive decision bias score indicates a preference for odor A; a negative decision bias score indicates a preference for odor B. Scores are given as mean ± SEM (n = 30–40 flies per data point). Shaded columns indicate bias scores under experimental conditions; columns without shading report bias scores under cross-control and temperature control conditions, and of parental control strains.

**Table S4, Related to Figures 4 and S4. Decision Bias Scores of *mb247-LexA:lexAop-shi$^{ts1}$* Flies and Parental Control Strains**

| | Odors | | Euclidean distance | Cosine distance | Decision bias (%) | | | | |
|---|---|---|---|---|---|---|---|---|---|
| | | | | | *mb247-LexA:lexAop-shi$^{ts1}$* | *mb247-LexA:lexAop-shi$^{ts1}$* trained at 25⁰C | *mb247-LexA:lexAop-shi$^{ts1}$* trained at 25⁰C | *mb247-LexA* trained at 25⁰C | *lexAop-shi$^{ts1}$* trained at 25⁰C |
| | Odor A | Odor B | CS | CS | untrained at 32⁰C | tested at 25⁰C | tested at 32⁰C | tested at 32⁰C | tested at 32⁰C |
| 1 | 2-Heptanone | Pentyl acetate | 66 | 0.02 | -7.8 ± 3.1 | 72.4 ± 4.7 | -9.9 ± 2.9 | 70.1 ± 1.9 | 66.7 ± 4.4 |
| 2 | 2-Heptanone | Butyl acetate | 126 | 0.08 | -3.8 ± 3.7 | 71.3 ± 4.2 | 3.8 ± 4.7 | 70.1 ± 3.3 | 64.5 ± 4.7 |
| 3 | Hexyl acetate | 2-Heptanone | 143 | 0.10 | 7.3 ± 2.0 | 75.8 ± 2.4 | 7.9 ± 2.7 | 80.3 ± 3.8 | 75.8 ± 5.0 |
| 4 | 1-Octanol | 2-Heptanone | 171 | 0.15 | -2.0 ± 2.8 | 69.4 ± 3.7 | -3.3 ± 3.8 | 75.6 ± 4.6 | 74.3 ± 4.7 |
| 5 | Isopentyl acetate | Ethyl butyrate | 188 | 0.17 | 6.8 ± 2.2 | 81.1 ± 2.5 | 7.0 ± 1.8 | 80.8 ± 2.4 | 80.4 ± 3.5 |
| 6 | 1-Octanol | 2-Pentanol | 192 | 0.19 | 10.3 ± 2.7 | 73.0 ± 4.3 | 13.9 ± 3.5 | 79.6 ± 2.2 | 76.3 ± 4.8 |
| 7 | 1-Octanol | 6-Methyl-5-hepten-2-one | 207 | 0.24 | -14.4 ± 4.9 | 69.6 ± 3.8 | -18.7 ± 4.2 | 69.1 ± 4.5 | 64.4 ± 3.3 |
| 8 | 1-Octanol | Isopentyl acetate | 233 | 0.28 | -27.3 ± 4.5 | 71.5 ± 4.6 | -23.2 ± 3.3 | 67.4 ± 4.0 | 72.1 ± 3.2 |
| 9 | 1-Octanol | Ethyl acetate | 255 | 0.37 | 29.3 ± 4.2 | 80.6 ± 3.2 | 32.6 ± 5.1 | 79.6 ± 3.5 | 78.9 ± 3.7 |
| 10 | 1-Octanol | Benzaldehyde | 289 | 0.46 | 49.2 ± .8 | 80.9 ±3 .2 | 43.4 ± 4.4 | 84.4 ± 3.3 | 83.9 ± 2.9 |

Decision bias scores of all odor pairs shown in Figures 4 and S4. Odor pairs are sorted according to the Euclidean distance between the predicted ePN activity vectors of the two odors. A positive decision bias score indicates a preference for odor A; a negative decision bias score indicates a preference for odor B. Scores are given as mean ± SEM (n = 30–60 flies per data point).

## Table S5, Related to Figures 6 and S6. Decision bias scores of *Mz699-GAL4:UAS-shi$^{ts1}$* Flies and Parental Control Strains

| | Odors | | Euclidean distance | Cosine distance | Decision bias (%) | | | |
|---|---|---|---|---|---|---|---|---|
| | Odor A | Odor B | CS | CS | *Mz699-GAL4:UAS-shi$^{ts1}$* 25$^0$C | *Mz699-GAL4:UAS-shi$^{ts1}$* 32$^0$C | *Mz699-GAL4* | *UAS-shi$^{ts1}$* |
| **1** | 1-Octanol | 1-Octanol | 0 | 0 | 0.7 ± 2.9 | 0.0 ± 1.6 | 1.2 ± 2.5 | 0.3 ± 2.0 |
| **2** | 2-Heptanone | Pentyl acetate | 66 | 0.02 | -7.5 ± 4.3 | -5.1 ± 1.9 | 0.6 ± 2.1 | -8.2 ± 2.2 |
| **3** | Butyl acetate | Isopentyl acetate | 72 | 0.02 | -10.0 ± 3.1 | -3.3 ± 1.0 | -9.7 ± 3.2 | -9.2 ± 1.6 |
| **4** | Isopentyl acetate | Isobutyl acetate | 107 | 0.05 | 9.0 ± 4.3 | 9.1 ± 2.4 | 6.5 ± 2.7 | 6.3 ± 1.4 |
| **5** | 2-Heptanone | Butyl acetate | 126 | 0.08 | 4.8 ± 2.2 | 1.3 ± 1.6 | 4.7 ± 2.0 | 8.1 ± 3.4 |
| **6** | Hexyl acetate | Pentyl acetate | 126 | 0.08 | 18.8 ± 2.7 | 8.8 ± 2.9 | 17.3 ± 2.1 | 6.1 ± 1.8 |
| **7** | 1-Octanol | Hexyl butyrate | 132 | 0.10 | 7.6 ± 2.4 | 5.4 ± 1.4 | 6.7 ± 3.3 | 9.1 ± 2.5 |
| **8** | Butyl acetate | Pentyl acetate | 139 | 0.09 | -7.2 ± 3.0 | -6.5 ± 1.7 | -13.0 ± 3.0 | -13.9 ± 2.6 |
| **9** | Hexyl acetate | 2-Heptanone | 143 | 0.10 | 3.0 ± 3.0 | 5.6 ± 5.8 | 4.1 ± 3.0 | 3.1 ± 2.5 |
| **10** | Isopentyl acetate | 2-Heptanone | 150 | 0.11 | 12.4 ± 5.0 | 6.1 ± 2.9 | 12.8 ± 5.5 | 15.9 ± 4.1 |
| **11** | Isopentyl acetate | 6-Methyl-5-hepten-2-one | 164 | 0.13 | 18.5 ± 7.5 | 4.5 ± 2.6 | 16.8 ± 4.6 | 15.9 ± 2.1 |
| **12** | 1-Octanol | 2-Heptanone | 171 | 0.15 | 2.0 ± 5.1 | 0.9 ± 2.2 | 4.7 ± 2.6 | -0.4 ± 4.1 |
| **13** | 2-Pentanol | 2-Heptanone | 171 | 0.14 | -16.0 ± 3.1 | 2.5 ± 3.0 | -13.0 ± 2.3 | -13.7 ± 2.5 |
| **14** | 1-Octanol | Hexyl acetate | 184 | 0.18 | -5.7 ± 2.7 | 1.7 ± 2.2 | -6.2 ± 3.1 | -9.2 ± 1.8 |
| **15** | Isopentyl acetate | Ethyl butyrate | 188 | 0.17 | 6.1 ± 5.3 | 4.8 ± 3.6 | 8.5 ± 2.9 | 1.0 ± 2.9 |
| **16** | 1-Octanol | 2-Pentanol | 192 | 0.19 | 10.1 ± 4.9 | -1.4 ± 2.7 | 12.5 ± 3.2 | 11.8 ± 4.9 |
| **17** | Hexyl acetate | Hexyl butyrate | 196 | 0.21 | 27.1 ± 2.7 | -1.8 ± 4.1 | 25.9 ± 4.2 | 19.1 ± 2.4 |
| **18** | 2-Pentanol | Ethyl acetate | 200 | 0.21 | 15.8 ± 4.4 | 3.8 ± 2.1 | 16.7 ± 3.6 | 13.1 ± 3.2 |
| **19** | 1-Octanol | 6-Methyl-5-hepten-2-one | 207 | 0.24 | -13.6 ± 5.3 | -6.4 ± 2.7 | -14.1 ± 3.4 | -17.1 ± 4.5 |
| **20** | Isopentyl acetate | 2-Pentanol | 208 | 0.21 | 24.8 ± 3.2 | 8.3 ± 3.1 | 21.1 ± 5.9 | 21.1 ± 5.5 |
| **21** | Acetophenone | Benzyl alcohol | 210 | 0.24 | -22.4 ± 3.3 | -8.5 ± 2.6 | -20.6 ± 3.6 | -23.7 ± 3.2 |
| **22** | Dimethyl sulfide | Ethyl octanoate | 229 | 0.33 | -13.8 ± 2.8 | -16.5 ± 2.2 | -14.3 ± 2.4 | -14.1 ± 3.0 |
| **23** | 1-Octanol | Isopentyl acetate | 233 | 0.28 | -25.4 ± 3.6 | -8.7 ± 1.7 | -20.4 ± 1.7 | -22.0 ± 3.4 |
| **24** | Isopentyl acetate | Ethyl acetate | 242 | 0.30 | 33.1 ± 6.8 | 30.1 ± 4.6 | 28.1 ± 6.6 | 27.2 ± 3.1 |
| **25** | Dimethyl sulfide | Hexanal | 242 | 0.34 | 26.1 ± 5.0 | 10.0 ± 8.5 | 33.2 ± 5.8 | 29.4 ± 2.4 |
| **26** | 2-Pentanol | 6-Methyl-5-hepten-2-one | 244 | 0.30 | -39.1 ± 3.4 | -28.1 ± 2.9 | -37.1 ± 4.0 | -34.2 ± 1.9 |
| **27** | Dimethyl sulfide | Nerol | 244 | 0.35 | -18.3 ± 2.9 | -5.9 ± 1.7 | -17.4 ± 3.2 | -19.7 ± 1.9 |
| **28** | Acetophenone | Linalool oxide | 245 | 0.33 | -45.5 ± 3.3 | -41.6 ± 2.8 | -36.0 ± 3.8 | -32.5 ± 3.7 |
| **29** | 1-Octanol | Ethyl acetate | 255 | 0.37 | 32.6 ± 5.3 | 28.3 ± 3.6 | 27.8 ± 2.0 | 34.2 ± 3.9 |
| **30** | 2-Pentanol | Benzaldehyde | 256 | 0.33 | 67.6 ± 3.3 | 59.4 ± 3.7 | 71.1 ± 4.7 | 67.1 ± 4.1 |
| **31** | Acetophenone | Diethyl succinate | 263 | 0.40 | -44.9 ± 2.9 | -35.6 ± 3.3 | -39.1 ± 2.4 | -43.8 ± 3.4 |
| **32** | Dimethyl sulfide | 1-Octanol | 268 | 0.44 | -21.9 ± 2.8 | -2.6 ± 2.8 | -19.0 ± 3.0 | -22.9 ± 4.1 |
| **33** | Linalool | Acetone | 271 | 0.42 | 36.9 ± 4.8 | 37.2 ± 3.4 | 33.7 ± 3.2 | 32.2 ± 2.6 |
| **34** | Dimethyl sulfide | Diethyl succinate | 272 | 0.46 | -27.4 ± 3.9 | -5.8 ± 4.1 | -39.5 ± 2.2 | -37.4 ± 3.8 |
| **35** | Isopentyl acetate | Benzaldehyde | 273 | 0.37 | 45.2 ± 5.2 | 43.4 ± 4.9 | 44.3 ± 5.8 | 46.2 ± 3.5 |
| **36** | Dimethyl sulfide | 6-Methyl-5-hepten-2-one | 284 | 0.46 | -34.7 ± 5.5 | -44.0 ± 3.2 | -42.2 ± 2.8 | -34.4 ± 4.0 |
| **37** | Acetophenone | Nerol | 286 | 0.45 | -41.3 ± 3.4 | -26.7 ± 3.4 | -47.3 ± 3.4 | -42.2 ± 2.1 |
| **38** | Ethyl benzoate | 1-Octanol | 287 | 0.50 | 3.3 ± 2.2 | 2.0 ± 1.7 | 5.9 ± 2.3 | 12.6 ± 2.5 |
| **39** | 1-Octanol | Benzaldehyde | 289 | 0.46 | 55.5 ± 5.8 | 48.9 ± 3.0 | 50.4 ± 2.9 | 47.2 ± 2.1 |
| **40** | Ethyl benzoate | Ethyl lactate | 289 | 0.49 | 28.4 ± 3.5 | 24.0 ± 3.5 | 19.7 ± 5.6 | 28.6 ± 4.7 |
| **41** | Acetophenone | 1-Octanol | 296 | 0.50 | -42.6 ± 3.4 | -35.4 ± 3.7 | -47.5 ± 3.0 | -48.6 ± 3.0 |
| **42** | Acetophenone | Hexyl acetate | 303 | 0.49 | -49.3 ± 3.5 | -41.8 ± 2.8 | -52.0 ± 2.9 | -41.2 ± 2.2 |
| **43** | Ethyl benzoate | 2-Pentanol | 314 | 0.54 | 45.2 ± 7.1 | 24.8 ± 2.8 | 46.2 ± 3.7 | 43.3 ± 3.5 |
| **44** | Dimethyl sulfide | Benzaldehyde | 315 | 0.56 | 44.9 ± 3.8 | 48.6 ± 3.9 | 42.7 ± 3.0 | 40.7 ± 2.8 |
| **45** | Geranyl acetate | 2-Pentanone | 316 | 0.57 | 32.6 ± 4.6 | 35.6 ± 4.0 | 35.8 ± 3.3 | 32.2 ± 3.2 |
| **46** | Pentyl acetate | Methyl salicylate | 317 | 0.56 | 33.0 ± 2.6 | 31.0 ± 3.8 | 33.0 ± 2.1 | 35.8 ± 3.7 |
| **47** | δ-Decalactone | Benzaldehyde | 318 | 0.60 | 39.5 ± 4.2 | 46.8 ± 3.1 | 47.2 ± 5.1 | 43.7 ± 3.6 |
| **48** | Hexyl acetate | Methyl salicylate | 326 | 0.64 | 33.9 ± 5.2 | 37.5 ± 3.8 | 40.0 ± 3.8 | 42.7 ± 4.0 |
| **49** | Dimethyl sulfide | Ethyl benzoate | 328 | 0.68 | -35.2 ± 4.3 | -30.2 ± 4.1 | -41.8 ± 2.3 | -27.8 ± 2.6 |
| **50** | Geranyl acetate | Benzaldehyde | 333 | 0.65 | 43.6 ± 4.9 | 47.1 ± 3.7 | 54.6 ± 4.3 | 39.8 ± 4.1 |
| **51** | 1-Butanol | Ethyl benzoate | 333 | 0.62 | -32.2 ± 2.7 | -3.2 ± 2.9 | -35.8 ± 3.9 | -39.0 ± 3.2 |

Decision bias scores of all odor pairs shown in Figures 6A, 6B, and S6. Odor pairs are sorted according to the Euclidean distance between the predicted ePN activity vectors of the two odors. A positive decision bias score indicates a preference for odor A; a negative decision bias score indicates a preference for odor B. Scores are given as mean ± SEM (n = 40–60 flies per data point).

**Table S6, Related to Figures 6 and S6. Decision Bias Scores of *Mz699-GAL4: UAS-vGAT-RNAi, UAS-GAD-RNAi* Flies and Parental Control Strains**

| | Odors | | Euclidean distance | Cosine distance | Decision bias (%) | | |
|---|---|---|---|---|---|---|---|
| | Odor A | Odor B | CS | CS | *tub-GAL80$^{ts}$, Mz699-GAL4: UAS-vGAT-RNAi ,UAS-GAD-RNAi* | *tub-GAL80$^{ts}$, UAS-vGAT-RNAi, UAS-GAD-RNAi* | *Mz699-GAL4* |
| **1** | Isopentyl acetate | 2-Heptanone | 150 | 0.11 | 7.5 ± 3.9 | 17.7 ± 1.9 | 12.8 ± 5.5 |
| **2** | Isopentyl acetate | 6-Methyl-5-hepten-2-one | 164 | 0.13 | 10.7 ± 2.9 | 20.9 ± 5.4 | 16.8 ± 4.6 |
| **3** | 1-Octanol | 2-Heptanone | 171 | 0.15 | -1.1 ± 3.5 | 0.4 ± 3.2 | 4.7 ± 2.6 |
| **4** | 2-Pentanol | 2-Heptanone | 171 | 0.14 | 0.6 ± 4.4 | -14.8 ± 2.7 | -13.0 ± 2.3 |
| **5** | 1-Octanol | 2-Pentanol | 192 | 0.19 | 2.2 ± 3.4 | 14.6 ± 9.8 | 12.5 ± 3.2 |
| **6** | 2-Pentanol | Ethyl acetate | 200 | 0.21 | 8.5 ± 4.9 | 11.3 ± 4.7 | 16.7 ± 3.6 |
| **7** | 1-Octanol | 6-Methyl-5-hepten-2-one | 207 | 0.24 | -6.5 ± 3.5 | -17.0 ± 2.6 | -14.1 ± 3.4 |
| **8** | Isopentyl acetate | 2-Pentanol | 208 | 0.21 | 7.4 ± 2.3 | 26.3 ± 7.2 | 21.1 ± 5.9 |
| **9** | 1-Octanol | Isopentyl acetate | 233 | 0.28 | -9.8 ± 3.7 | -31.4 ± 4.2 | -20.4 ± 1.7 |
| **10** | Isopentyl acetate | Ethyl acetate | 242 | 0.30 | 28.8 ± 3.5 | 29.4 ± 2.8 | 28.1 ± 6.6 |
| **11** | 2-Pentanol | 6-Methyl-5-Hepten-2-one | 244 | 0.30 | -28.0 ± 3.5 | -38.4 ± 4.1 | -37.1 ± 4.0 |
| **12** | 1-Octanol | Ethyl acetate | 255 | 0.37 | 26.0 ± 3.7 | 33.8 ± 8.7 | 27.8 ± 2.0 |
| **13** | 2-Pentanol | Benzaldehyde | 256 | 0.33 | 43.9 ± 2.9 | 57.9 ± 4.5 | 71.1 ± 4.7 |
| **14** | Isopentyl acetate | Benzaldehyde | 273 | 0.37 | 41.4 ± 3.5 | 48.3 ± 3.5 | 44.3 ± 5.8 |
| **15** | 1-Octanol | Benzaldehyde | 289 | 0.46 | 48.0 ± 3.0 | 54.5 ± 4.6 | 50.4 ± 2.9 |

Decision bias scores of all odor pairs shown in Figures 6C and S6. Odor pairs are sorted according to the Euclidean distance between the predicted ePN activity vectors of the two odors. A positive decision bias score indicates a preference for odor A; a negative decision bias score indicates a preference for odor B. Scores are given as mean ± SEM (n = 40–60 flies per data point).

## SUPPLEMENTAL EXPERIMENTAL PROCEDURES

### *Fly Strains*

Experimental animals carried transgenes over Canton-S chromosomes where possible to minimize genetic differences between strains. Effector transgenes encoded fluorescent markers for neuronal membranes (*UAS-mCD8-GFP*, Lee and Luo, 1999), dendrites (*UAS-DenMark*, Nicolai et al., 2010), or synaptic terminals (*UAS-$^{GFP}$DSyd-1*, Owald et al., 2010); sensors for intracellular calcium levels (*UAS-GCaMP3*, Tian et al., 2009) or synaptic release (*UAS-spH*, Miesenböck et al., 1998; Ng et al., 2002; or *QUAS-spH,* see below); hairpin constructs (Dietzl et al., 2007) for interference with the expression of GABA biosynthetic enzymes and transporters (*tub-GAL80$^{ts}$;UAS-vGAT-RNAi;UAS-GAD-RNAi)*; and temperature-dependent blockers of synaptic transmission (*UAS-shi$^{ts1}$*, *lexAop-shi$^{ts1}$*, Kitamoto, 2001) or activators of neural activity (*UAS-dTRPA1*, Hamada et al., 2008). Driver lines directed expression to ePNs (*GH146-GAL4*, Stocker et al., 1997; *GH146-QF*, Potter et al., 2010; *NP3062-GAL4*, Olsen et al., 2010; Tanaka et al., 2012; *NP1579-GAL4*, Tanaka et al., 2012), iPNs (*Mz699-GAL4,* Lai et al., 2008), or KCs (*mb247-GAL4*, *mb247-LexA*; Pitman et al., 2011; Zars et al., 2000).

To create *pQUAST-spH*, the spH coding sequence was excised from *pUAST-spH* (Miesenböck et al., 1998; Ng et al., 2002) and ligated to pQUAST (Potter et al., 2010); *QUAS-spH* transgenic flies were generated by random P-element insertion. cDNA encoding GCaMP3 (Tian et al., 2009) was ligated to pCa4B2G (Hamada et al., 2008; Markstein et al., 2008) and targeted to attP2 and attP16 landing sites (Genetic Services, Inc.).

### *Behavioral Analysis*

Flies were housed individually in clear polycarbonate chambers with printed circuit boards (PCBs) serving as floors and ceilings. Solid-state relays (Fairchild HSR312L) connected the PCBs to a 60 V source. For electric shock reinforcement, the relays were activated for 1.25 s at a repetition rate of 0.2 Hz during a 1 min odor presentation (Claridge-Chang et al., 2009).

Flow-controlled carrier air (2.7 l/min; CMOSens PerformanceLine, Sensirion), was combined with flow-controlled stimulus streams (0.3 l/min) drawn through vials filled with liquid odorants at 10-fold dilution in mineral oil. Mixing carrier and stimulus streams resulted in a further 10-fold dilution of the odorants, whose final airborne concentrations (at $10^{-2}$ dilution) were measured daily with a ppbRAE photoionization detector. The air/odor streams were split between 20 chambers, yielding a flow rate of 0.15 l/min per half-chamber. Air/odor streams converged from the left and right chamber halves at a central choice zone. A stack of 20 chambers was backlit by 940 nm LEDs (TSAL6100, Vishay) and imaged by a Stingray F080B CCD camera (Allied Vision Technologies) equipped with a Computar M1614 lens. The apparatus was operated in a temperature-controlled incubator (Sanyo MIR-154) maintained at 25 or 32 °C, as indicated.

A virtual instrument written in LabVIEW 7.1 (National Instruments) extracted fly position data from video images and controlled the delivery of odors and electric shocks. Data were analyzed in MATLAB 2009b (The MathWorks), SigmaPlot 12.5 (Systat Software), and Prism 6 (GraphPad).

***Functional Imaging***

Two-photon fluorescence was excited with 140 fs pulses of light centered at 910 nm (Chameleon Ultra II, Coherent). The excitation laser was attenuated by a Pockels cell (Conoptics 302RM) and coupled to the scan engine of a Movable Objective Microscope (Sutter Instruments) equipped with a Zeiss 20×, 1.0 NA W-Plan-Apochromat objective. GaAsP photomultiplier currents (Hamamatsu Photonics H10770PA-40 SEL) were amplified (Laser Components HCA-4M-500K-C) and passed through a custom-designed integrator circuit. The microscope was controlled through MPScope 2.0 via a PCI-6110 DAQ board (National Instruments).

Images in Analyze format were motion-corrected by maximizing the pixel-by-pixel correlation with a reference frame. Pseudocolored activity maps were generated by comparing the mean difference in fluorescence intensity during an

odor pulse (ΔF) with the average intensity over a 10 s pre-stimulus interval. If ΔF of a pixel was less than twice the standard deviation of the intensity of that pixel during the pre-stimulus interval, the pixel was considered unresponsive. Activity maps were smoothed with a Gaussian filter for display purposes, but not for statistical similarity analyses. These analyses were performed after aligning the activity maps of different odors by maximizing correlations in baseline fluorescence and then calculating pairwise Pearson correlation coefficients.

***Structural Imaging***

To visualize native GFP or GCaMP3 fluorescence, female flies were dissected 4–10 days after eclosion. Brains were recovered into an ice-cold solution of 4% (w/v) paraformaldehyde in PBS (1.86 mM $NaH_2PO_4$, 8.41 mM $Na_2HPO_4$, 175 mM NaCl) and fixed for 60–120 min at room temperature under vacuum. Samples were washed for 3×10 min in PBS containing 0.1% (v/v) Triton-X100 (PBT) and twice in PBS before mounting in Vectashield (Vector Labs). For immunostainings, GABA was detected with rabbit anti-GABA antibody (A2052, Sigma-Aldrich, 1:100) and goat anti-rabbit Alexa 546 conjugate (Jackson ImmunoResearch, 1:200); GFP was enhanced with mouse anti-GFP IgG2a (A11120, Invitrogen, 1:200) and goat anti-mouse Alexa 488 conjugate (Jackson ImmunoResearch, 1:200). Primary antisera were applied for 3 days and secondary antisera for 2 days in PBT at 4 ˚C. A nuclear marker (TOTO-3, Invitrogen, 1:1000) was added for 1h before washing, followed by embedding in Vectashield. Images were collected on a Leica TCS SP5 confocal microscope and processed in Amira 5.3 (Visualization Sciences Group).

## Supplemental References


Claridge-Chang, A., Roorda, R.D., Vrontou, E., Sjulson, L., Li, H., Hirsh, J., and Miesenböck, G. (2009). Writing memories with light-addressable reinforcement circuitry. Cell *139*, 405–415.

Dietzl, G., Chen, D., Schnorrer, F., Su, K.C., Barinova, Y., Fellner, M., Gasser, B., Kinsey, K., Oppel, S., Scheiblauer, S., et al. (2007). A genome-wide transgenic RNAi library for conditional gene inactivation in Drosophila. Nature *448*, 151–156.

Hallem, E.A., and Carlson, J.R. (2006). Coding of odors by a receptor repertoire. Cell *125*, 143–160.

Hallem, E.A., Ho, M.G., and Carlson, J.R. (2004). The molecular basis of odor coding in the Drosophila antenna. Cell *117*, 965–979.

Hamada, F.N., Rosenzweig, M., Kang, K., Pulver, S.R., Ghezzi, A., Jegla, T.J., and Garrity, P.A. (2008). An internal thermal sensor controlling temperature preference in Drosophila. Nature *454*, 217–220.

Kitamoto, T. (2001). Conditional modification of behavior in Drosophila by targeted expression of a temperature-sensitive shibire allele in defined neurons. J Neurobiol *47*, 81–92.

Lai, S.L., Awasaki, T., Ito, K., and Lee, T. (2008). Clonal analysis of Drosophila antennal lobe neurons: diverse neuronal architectures in the lateral neuroblast lineage. Development (Cambridge, England) *135*, 2883–2893.

Lee, T., and Luo, L. (1999). Mosaic analysis with a repressible cell marker for studies of gene function in neuronal morphogenesis. Neuron *22*, 451–61.

Markstein, M., Pitsouli, C., Villalta, C., Celniker, S.E., and Perrimon, N. (2008). Exploiting position effects and the gypsy retrovirus insulator to engineer precisely expressed transgenes. Nat Genet *40*, 476–483.

Miesenböck, G., De Angelis, D.A., and Rothman, J.E. (1998). Visualizing secretion and synaptic transmission with pH-sensitive green fluorescent proteins. Nature *394*, 192–195.

Ng, M., Roorda, R.D., Lima, S.Q., Zemelman, B.V., Morcillo, P., and Miesenböck, G. (2002). Transmission of olfactory information between three populations of neurons in the antennal lobe of the fly. Neuron *36*, 463–474.

Nicolai, L.J., Ramaekers, A., Raemaekers, T., Drozdzecki, A., Mauss, A.S., Yan, J., Landgraf, M., Annaert, W., and Hassan, B.A. (2010). Genetically encoded dendritic marker sheds light on neuronal connectivity in Drosophila. Proc Natl Acad Sci USA *107*, 20553–20558.

Olsen, S.R., Bhandawat, V., and Wilson, R.I. (2010). Divisive normalization in

olfactory population codes. Neuron *66*, 287–299.

Owald, D., Fouquet, W., Schmidt, M., Wichmann, C., Mertel, S., Depner, H., Christiansen, F., Zube, C., Quentin, C., Korner, J., et al. (2010). A Syd-1 homologue regulates pre- and postsynaptic maturation in Drosophila. J Cell Biol *188*, 565–579.

Pitman, J.L., Huetteroth, W., Burke, C.J., Krashes, M.J., Lai, S.L., Lee, T., and Waddell, S. (2011). A pair of inhibitory neurons are required to sustain labile memory in the Drosophila mushroom body. Curr Biol *21*, 855–861.

Potter, C.J., Tasic, B., Russler, E.V., Liang, L., and Luo, L. (2010). The Q system: a repressible binary system for transgene expression, lineage tracing, and mosaic analysis. Cell *141*, 536–548.

Stocker, R.F., Heimbeck, G., Gendre, N., and de Belle, J.S. (1997). Neuroblast ablation in Drosophila P[GAL4] lines reveals origins of olfactory interneurons. J Neurobiol *32*, 443–456.

Tanaka, N.K., Endo, K., and Ito, K. (2012). The organization of antennal lobe-associated neurons in the adult Drosophila melanogaster brain. J Comp Neurol.

Tian, L., Hires, S.A., Mao, T., Huber, D., Chiappe, M.E., Chalasani, S.H., Petreanu, L., Akerboom, J., McKinney, S.A., Schreiter, E.R., et al. (2009). Imaging neural activity in worms, flies and mice with improved GCaMP calcium indicators. Nat Methods *6*, 875–881.

Zars, T., Fischer, M., Schulz, R., and Heisenberg, M. (2000). Localization of a short-term memory in Drosophila. Science *288*, 672–5.

## Supplemental Movie Legends

**Movie S1, Related to Figure 5. Dendritic and Axonal Arborizations of Neurons Labeled by the *Mz699-GAL4* Driver Line**

Stack of 67 confocal sections (1.5 μm) through the vlpr of a fly carrying *Mz699-GAL4:UAS-mCD8-GFP* transgenes. $^{GFP}$DSyd-1 expression (cyan) labels presynaptic axonal terminals. DenMark expression (magenta) labels putative dendritic regions.

**Movie S2, Related to Figure 5. GABAergic iPNs**

Stack of 60 confocal sections (1.5 μm) through the antennal lobe of a fly carrying *Mz699-GAL4:UAS-mCD8-GFP* transgenes, after immunostaining against GABA (magenta) and GFP (cyan) and counterstaining with the nuclear marker TOTO-3 (yellow).

**Movie S3, Related to Figure 5. vlpr Neurons**

Stack of 56 confocal sections (1.5 μm) through the vlpr of a fly carrying *Mz699-GAL4:UAS-mCD8-GFP* transgenes, after immunostaining against GABA (magenta) and GFP (cyan) and counterstaining with the nuclear marker TOTO-3 (yellow).
