# Supplementary figures and images for: Odor Discrimination in Drosophila: From Neural Population Codes to Behavior
Source: Neuron. 2013 Sep 4;79(5):932–44. doi: 10.1016/j.neuron.2013.08.006 (PMC3765961; doi:10.1016/j.neuron.2013.08.006)

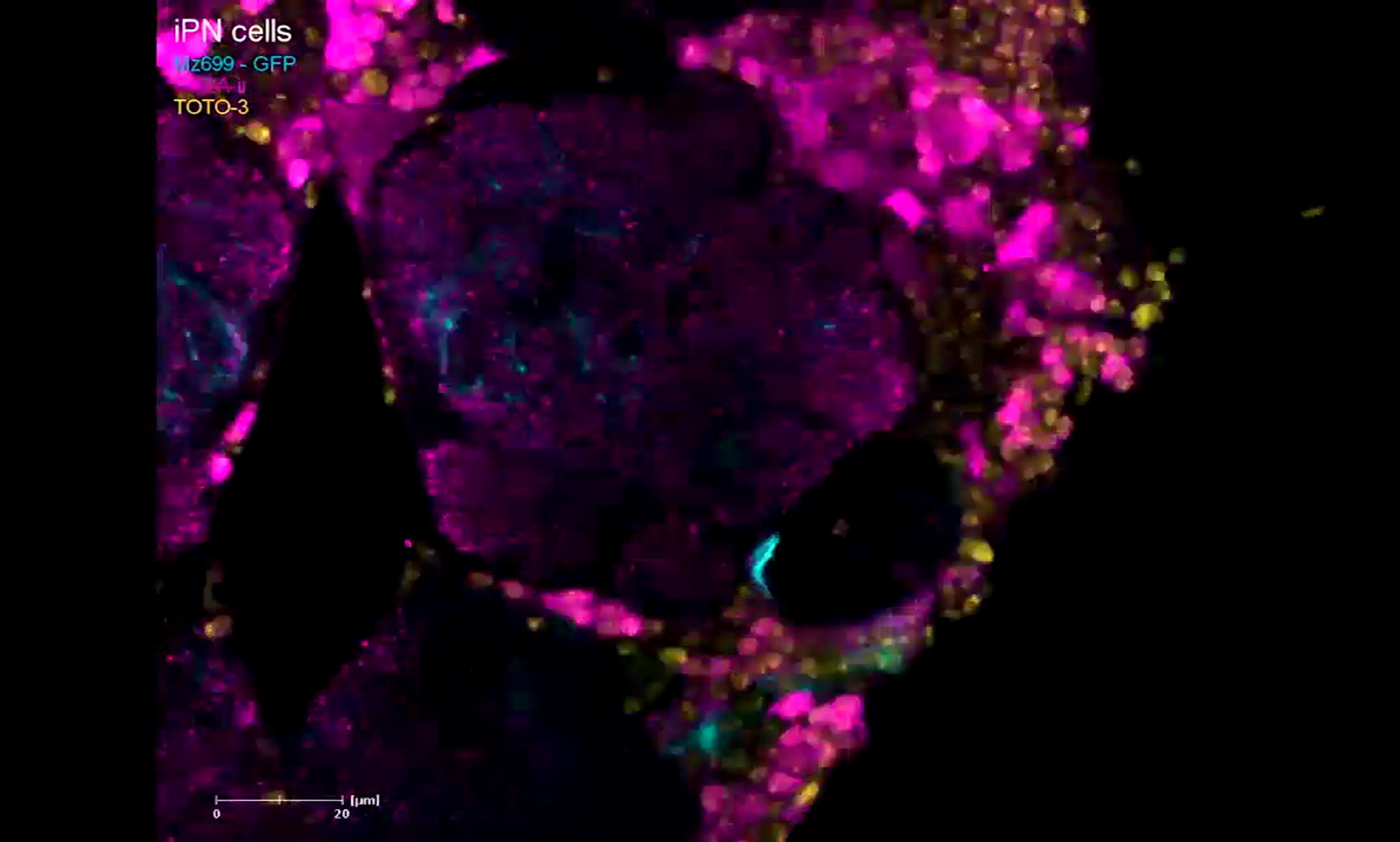

Supplement: Movie S2. GABAergic iPNs, Related to Figure 5 — Stack of 60 confocal sections (1.5 μm) through the antennal lobe of a fly carrying Mz699-GAL4:UAS-mCD8-GFP transgenes after immunostaining against GABA (magenta) and GFP (cyan) and counterstaining with the nuclear marker TOTO-3 (yellow). [file mmc3.jpg]

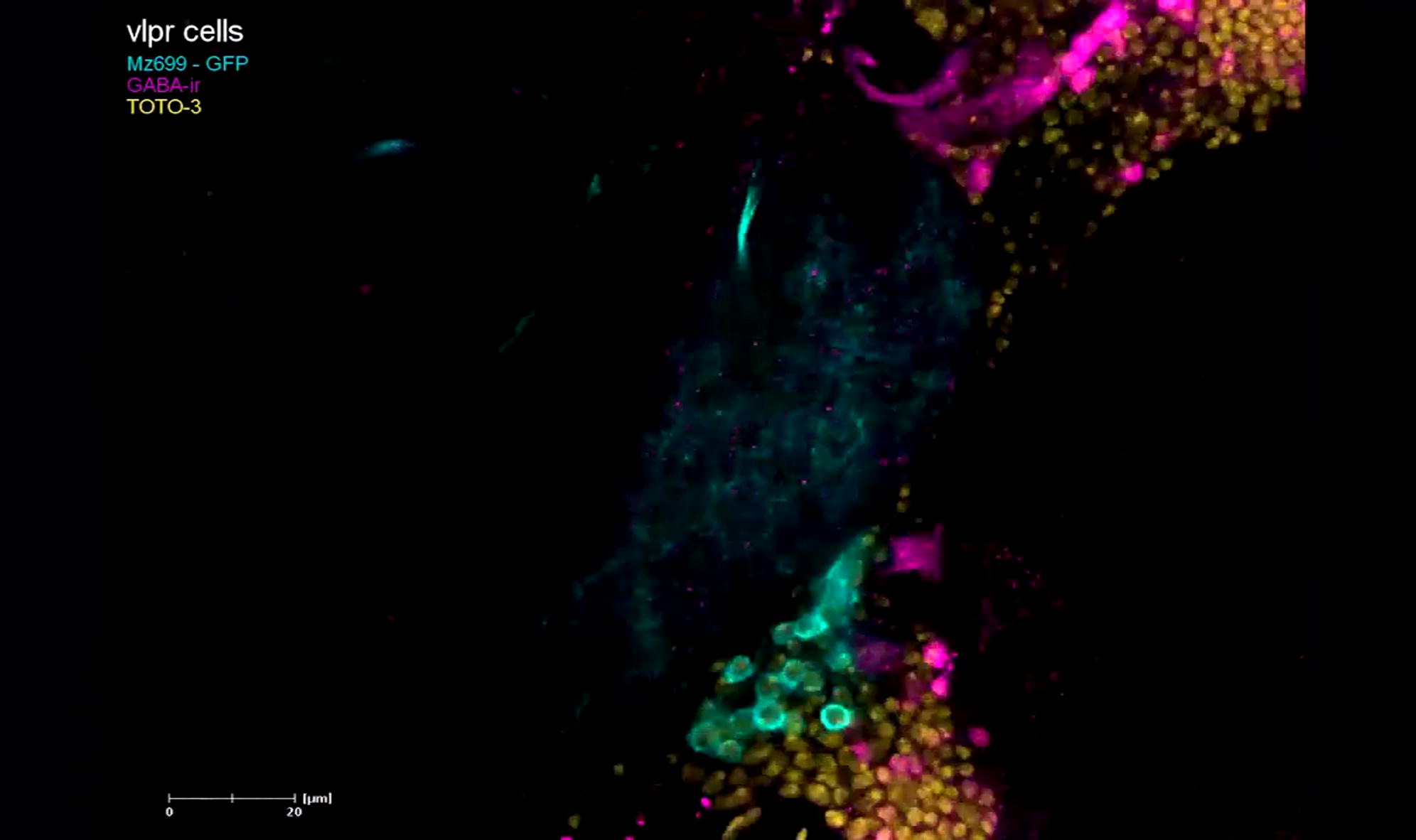

Supplement: Movie S3. vlpr Neurons, Related to Figure 5 — Stack of 56 confocal sections (1.5 μm) through the vlpr of a fly carrying Mz699-GAL4:UAS-mCD8-GFP transgenes after immunostaining against GABA (magenta) and GFP (cyan) and counterstaining with the nuclear marker TOTO-3 (yellow). [file mmc4.jpg]
